# Prediction of the Carcinogenic Potential of Human Pharmaceuticals Using Repeated Dose Toxicity Data and Their Pharmacological Properties

**DOI:** 10.3389/fmed.2016.00045

**Published:** 2016-10-14

**Authors:** Jan Willem van der Laan, Wenny H. W. Buitenhuis, Laura Wagenaar, Ans E. M. F. Soffers, Eugene P. van Someren, Cyrille A. M. Krul, Ruud A. Woutersen

**Affiliations:** ^1^Medicines Evaluation Board, Utrecht, Netherlands; ^2^Division of Toxicology, Leiden Academic Center for Drug Research, Leiden, Netherlands; ^3^Veterinary Faculty, IRAS, Utrecht, Netherlands; ^4^Division of Toxicology, Wageningen University and Research Centre, Wageningen, Netherlands; ^5^TNO Innovation for Life, Zeist, Netherlands

**Keywords:** carcinogenicity, pharmacology, human pharmaceuticals, histopathology, predictivity

## Abstract

In an exercise designed to reduce animal use, we analyzed the results of rat subchronic toxicity studies from 289 pharmaceutical compounds with the aim to predict the tumor outcome of carcinogenicity studies in this species. The results were obtained from the assessment reports available at the Medicines Evaluation Board of the Netherlands for 289 pharmaceutical compounds that had been shown to be non-genotoxic. One hundred forty-three of the 239 compounds not inducing putative preneoplastic lesions in the subchronic study did not induce tumors in the carcinogenicity study [true negatives (TNs)], whereas 96 compounds were categorized as false negatives (FNs) because tumors were observed in the carcinogenicity study. Of the remaining 50 compounds, 31 showed preneoplastic lesions in the subchronic study and tumors in the carcinogenicity study [true positives (TPs)], and 19 only showed preneoplastic lesions in subchronic studies but no tumors in the carcinogenicity study [false positives (FPs)]. In addition, we then re-assessed the prediction of the tumor outcome by integrating the pharmacological properties of these compounds. These pharmacological properties were evaluated with respect to the presence or absence of a direct or indirect proliferative action. We found support for the absence of cellular proliferation for 204 compounds (TN). For 67 compounds, the presence of cellular hyperplasia as evidence for proliferative action could be found (TP). Therefore, this approach resulted in an ability to predict non-carcinogens at a success rate of 92% and the ability to detect carcinogens at 98%. The combined evaluation of pharmacological and histopathological endpoints eventually led to only 18 unknown outcomes (17 categorized as FN and 1 as FP), thereby enhancing both the negative and positive predictivity of an evaluation based upon histopathological evaluation only. The data show the added value of a consideration of the pharmacological properties of compounds in relation to potential class effects, both in the negative and positive direction. A high negative and a high positive predictivity will both result in waiving the need for conducting 2-year rat carcinogenicity studies, if this is accepted by Regulatory Authorities, which will save large numbers of animals and reduce drug development costs and time.

## Introduction

Specific regulatory requirements for carcinogenicity assessment of new pharmaceuticals are described in International Conference (now Council) on Harmonisation (ICH) guidance documents, i.e., ICH Guidelines M3(R2) (1), S1A (2), S1B (3), S1C(R2) (4), S2 (5), and S6(R1) (6).

Carcinogenicity studies are generally required for new pharmaceuticals that would be administered for 6 months or longer, or in a frequent and intermittent manner. In general, a 2-year rat study, plus either an 18 (or 24)-month mouse study or an alternative 6- or 9-month study in transgenic mice, is requested for such compounds.

The current 2-year rodent carcinogenicity study design used since the mid-1960s has been the regulatory standard in the safety assessment of humans. These carcinogenicity studies are expensive and time-consuming animal tests required for the safety assessment of pharmaceutical compounds. However, there is considerable scientific doubt about the reliability of the rat bioassay. Too many compounds are positively enhancing a tumor response in these studies, which may simply be due to the long-term exposure of the animals to rather high doses of the test compound (7–9) rather than a true carcinogenic effect. Therefore, there is a continued and increasingly need to justify the 2-year rodent bioassay in an attempt to reduce animal numbers, time, and costs (10, 11). For this reason, pharmaceutical companies and regulatory bodies are aiming to find an alternative approach.

In light of concerns raised about the predictability of *in vivo* studies in general and the push for refinement, reduction, and replacement of animal studies, it is strongly recommended to re-evaluate the suitability of the 2-year rodent bioassay as the best approach to predict human disease (12–21).

We have undertaken a retrospective study of pharmaceuticals with available rodent subchronic (3- to 6-month studies) and carcinogenicity data to test the hypothesis that it is possible to replace the current 2-year bioassay with a weight-of-evidence approach, i.e., by using evidence from all the non-clinical data available at the stage of development of a compound, usually at the end of Clinical Phase II, when a decision for conducting a 2-year carcinogenicity study is usually taken. Data that contribute are the results of the subchronic toxicity studies, in combination with genotoxicity data and knowledge of pharmacodynamic properties relating to the mode of action.

Positive *in vivo* genotoxicity tests are generally considered as indicative for a carcinogenic potency of a compound. Under REACH (22), classification as a mutagen category 1A or 1B allows a waiving of the carcinogenicity study, since the default presumption is that a genotoxic mechanism for carcinogenicity is likely. The same is true for human pharmaceuticals, where the ICH S1A guideline indicates that in case of positive genotoxicity, no lifetime carcinogenicity studies are expected. Positivity in an assay for DNA reactivity will usually also preclude further development (23), unless the risk for genotoxicity is acceptable in view of the benefit of the compound.

Jacobs (24) examined the data from 13-week rat toxicity studies for the prediction of carcinogenicity outcome using 60 pharmaceutical compounds. The data were obtained from a USA Food and Drug Administration (FDA) database. She concluded that various short-term indicators of carcinogenicity, such as hyperplasia, hypertrophy, greater organ weight, tissue degeneration or atrophy, and mineralization in a tissue, did not always result in tumors in that tissue, although (some of) these indicators are considered signs of potential carcinogenicity. The tissues examined were limited to the liver, kidneys, mammary glands, adrenals, urinary bladder, and lung.

Reddy et al. (25) confirmed the conclusion of Jacobs (24) with a different dataset. They used a “whole animal response” instead of individual tissues, testing the hypothesis that evidence of absence of putative preneoplastic lesions in any tissue may accurately predict a compound’s lack of carcinogenic potential. In their view, the presence of treatment-related putative preneoplastic histopathological lesions is not a definitive indicator of a tumorigenic potential of a compound, but rather requires that a 24-month rat carcinogenicity study has to be run and all tissues and organs should be collected and examined.

Sistare et al. (16) further evaluated the predictivity of histopathological findings, considering risk factors for rat neoplasia (hypertrophy, hyperplasia, metaplasia, cell proliferation, foci of cellular alteration, and inflammation accompanied by recurrent cell necrosis and repair) that were observed microscopically in 6-month rat toxicity studies with the tumor outcomes in rat 2-year carcinogenicity studies for 182 pharmaceuticals derived from 13 pharmaceutical companies. They concluded that the absence, rather than the presence, of the aforementioned putative preneoplastic histopathological changes in rats was a reliable predictor of tumor outcome in the corresponding tissue. The authors proposed that compounds demonstrating no genotoxicity, no evidence for hormonal mechanisms, and no histopathological changes pointing to a risk factor for rat neoplasia in any tissue (called NEGCARC approach) are considered rat non-carcinogens and can be exempted from the requirement for testing in a 2-year carcinogenicity study.

Assessors from EU Regulatory Authorities on human pharmaceuticals started to evaluate this approach and this set of data by emphasizing the consideration of the pharmacological properties of each compound and relating these properties to the outcome of the rat carcinogenicity study. The dataset was extended with data from FDA and the Japanese Pharmaceutical Manufacturer’s Association, increasing the number of compounds to 255 (26). Pharmacological properties appeared to be well associated with the outcome of the rat carcinogenicity study, both in a positive and negative direction. Classes such as β_2_-agonists and dopamine D_2_ antagonists were rather strongly associated with induction of mesovarian leiomyoma and mammary gland tumors, respectively (26). In addition, compounds inducing liver-associated pathology appear to be important in predicting tumors in organs such as liver, thyroid, and testis. The enzymes responsible for metabolism are also important factors in this respect.

The present retrospective study is intended to gather information independently from the dataset of Sistare et al. (16) to further test the hypothesis that a weight-of-evidence approach is possible in predicting the carcinogenic potential of human pharmaceuticals based upon histopathological and pharmacological properties. We have used a more restrictive approach in the definition of putative preneoplastic changes as compared with Sistare et al. (16), which will be further discussed below.

## Materials and Methods

### Selection of Compounds

The rat subchronic toxicity and chronic carcinogenicity data used for this evaluation came from the assessment reports that are available at the Medicines Evaluation Board of the Netherlands. The compounds and studies that were included were those in the paper of Van Oosterhout et al. (27), which reviewed all of the carcinogenicity studies from 1979 when GLP was introduced, although it was not possible to retrieve all of the studies covered in that paper, those submitted for marketing authorization in the Netherlands between 1995 and 2004, and those authorized *via* the EU centralized procedures between 2004 and 2014. The criteria used to identify valid pairs of rat subchronic (3- and/or 6-month) studies and 2-year carcinogenicity studies were as described below.

### ToxRefDB Database Structure

We have downloaded an empty version of ToxRefDB from the website of the US Environment Protection Agency (EPA), and we have used the structured organization to summarize all the data available for the carcinogenicity studies. We started our assessment based on the assessment report, but in cases where there was a lack of detail, we used the non-clinical expert report, non-clinical overview, or other parts from the dossier available on microfilm or electronically.

### Comparison of Protocols of Subchronic Studies with Carcinogenicity Studies

Although similar criteria regarding overlap of dose range have been applied as previously reported by Reddy et al. (25) and Sistare et al. (16) for inclusion of the studies, we have included all compound datasets available, as the impact of the pharmacological properties is expected to be largely independent of the matching of doses.

The database contained the dose levels used in the subchronic and carcinogenicity studies and the effects of the pharmaceuticals on body weight, organ weight, histopathology of a large set of organs and tissues, and results of genotoxicity tests *in vitro* and *in vivo*.

## Criteria for Positive Genotoxicity Test Result

Genotoxicity is usually tested with a battery of genotoxicity assays, as detailed in the ICH guidelines S2A and 2B revised in S2R2 (5). The outcome of these tests was taken from the assessment reports and was not re-assessed.

## Compounds Classification and Description of Study Outcomes

The pharmaceuticals were classified for pharmacology, and subsequently, the histopathological outcome of the studies has been described. The results of genotoxicity studies were included to cover similar criteria as used by Sistare et al. (16).

### Pharmacological Criteria

The pharmacotherapeutic areas were based on target organs, such as central nervous system (CNS), cardiovascular system (CVS), respiratory system (RS), metabolic system (MB), hormonal system (HM), gastrointestinal system (GIS), immunological system (IS), and antimicrobials (AM) (divided into antibacterials, antimalarials, antivirals, antifungals, and remaining compounds). Each pharmacotherapeutic category was subdivided into classes according to the primary drug target of the compounds in accordance with Stefansdottir et al. (28). Small molecules may exert additional pharmacological activity at a higher dose, which is termed secondary pharmacodynamics and could, as described by Keiser et al. (29), be responsible for the carcinogenic response.

### Histopathological Criteria

Positive histopathology observations were scored if any of the selected histopathological changes were reported as being increased in the subchronic toxicity studies, i.e., cellular hypertrophy, cellular hyperplasia, presence of altered hyperplastic foci of cellular alteration (atypical) cell foci (basophilic; acidophilic foci), cellular proliferation, and dysplasia. Furthermore, any changes in body weights and organ weights changes were noted.

The compounds were scored as negative for histopathological evidence of potential preneoplasia when the aforementioned histopathological changes were absent or not considered as being increased by treatment. All incidences reported to be higher than the controls were considered positive; with no access to all of the original study reports, no attempts were made to re-evaluate them in terms of whether or not they were statistically significant. When the histopathological changes were incorporated in the database, they were considered to be related to treatment and significantly increased.

We have scored greater organ weights, hypertrophy, and hyperplastic findings separately in Table 1. Furthermore, we have listed the tumors observed by describing the organ system and the histopathological appearance.

**Table 1:** **Summary of the observations in the subchronic and carcinogenicity studies**.

#	Mode of action	Cat His	Cat Ph.	Fin Cat.	Weight	Subchronic		Carcinogenicity
						HT	HP	
231	AB, fluoroquinolone	FN	NT	FN	col; kid	–	–	hsyst leu
244	AB, fluoroquinolone	FN	NT	FN	–	–	–	pan tu
263	AB, fluoroquinolone	FN	NT	FN	ce; hrt; li; spl; adr; ova	–	–	kid ac
226	AF, conazole derivative	FN	NT	FN	li	li	–	li ad
236	AF, conazole derivative	FN	NT	FN	adr; li; hrt; kid; thy; lu; spl; pan; br; gon; ova	adr	–	soft t sar
279	AF, remaining, allylamine derivative	FN	NT	FN	hrt; adr	–	–	tes tu; li ad; li ac
246	AV, CCR5 receptor antagonist	FN	NT	FN	–	thyr	–	thyr ad
218	AV, hepatitis B-inhibitor	FN	NT	FN	–	–	–	pan ad; pan ac; li ad; li ac; zymgl ca; br gli
220	CNS, 5HT_2_ antagonist	FN	NC	FN	–	–	–	li ad
230	CNS, remaining, α_2_-delta agonist	FN	NC	FN	–	–	–	pan ac; pan ad; tes ad; ut polyp
251	CNS, remaining, antidepressant	FN	NC	FN	–	li; thyr	–	thyr ac; mam ca; li ad; li ac
217	CNS, remaining, COMT-inhibitor	FN	NC	FN	adr	–	–	kid ad; kid ac
229	CVS, loop diuretic	FN	NC	FN	–	–	–	thyr ad; pit ad
253	CVS, loop diuretic	FN	NC	FN	–	–	–	tes ad; ut ac
284	CVS, loop diuretic	FN	NC	FN	–	–	–	kid ac; kid ad
282	CVS, platelet aggregation inhibitor	FN	NC	FN	–	li	–	thyr ad; adr bpha; ut ac; li ad; ova ad; mam ad
242	IS, remaining, imidazothiazole derivative	FN	NC	FN	–	–	–	pit ad
206	AI, COX2 inhibitor	FN	TN	TN	li	–	–	li ac
222	AI, COX2 inhibitor	FN	TN	TN	–	li; thyr	–	thyr ad; li ad
260	AI, NSAID	FN	TN	TN	–	–	–	tes ad;
234	BM, remaining, isoflavone	FN	TN^a^	TN	–	–	–	pit ad; li ad
277	CNS, 5-HT_1b/d_ agonist	FN	TN	TN	–	–	–	adr bpha; tes ad
239	CNS, antiepileptic, Na-channel blocker	FN	TN	TN	adr; pit; kid; li	–	–	adr bpha
204	CNS, benzodiazepine	FN	TN	TN	–	–	–	thyr ad; thym lymph; ut schwan
248	CNS, benzodiazepine	FN	TN	TN	–	li	–	thyr ad
205	CNS, opioid, μ-agonist	FN	TN	TN	–	–	–	tes tu; hsyst leu
197	CNS, remaining, melatonin receptor agonist	FN	TN^a^	TN	–	–	–	li ad; li ac
223	CNS, remaining, NMDA-antagonist	FN	TN^a^	TN	–	–	–	tes ad
261	CNS, remaining, nootropic drug	FN	TN^a^	TN	–	–	–	adr bpha
250	CNS, SNRI	FN	TN	TN	–	li	–	thyr ad
276	CNS, SNRI	FN	TN	TN	kid	–	–	tes ad
262	CNS, SSRI	FN	TN	TN	li	–	–	ln lymph
208	CVS, ACE inhibitor	FN	TN	TN	kid; li	kid	–	tes tu
266	CVS, ACE inhibitor	FN	TN	TN	–	–	–	thyr ac
271	CVS, ACE inhibitor	FN	TN	TN	–	–	–	kid ad
285	CVS, ACE inhibitor	FN	TN	TN	–	–	–	mam fad
233	CVS, ACE inhibitor	FN	TN	TN	kid	–	–	thyr ad; ut polyp
249	CVS, α_1_ agonist	FN	TN	TN	–	–	–	tes ad
289	CVS, anticoagulant	FN	TN	TN	–	–	–	pan ad/ca
203	CVS, β antagonist	FN	TN	TN	tes; adr; li	–	–	pit tu
219	CVS, β antagonist	FN	TN	TN	kid	–	–	skin SCP
243	CVS, β antagonist	FN	TN	TN	thyr; li; adr; kid	–	–	li ad
255	CVS, β antagonist,	FN	TN	TN	–	–	–	spl bhaem
200	CVS, calcium antagonist	FN	TN	TN	–	–	–	ut polyp
235	CVS, calcium antagonist	FN	TN	TN	–	–	–	tes ad
237	CVS, calcium antagonist	FN	TN	TN	ova	–	–	tes ad
240	CVS, calcium antagonist	FN	TN	TN	–	adr	–	mam fad; pit ad
256	CVS, calcium antagonist	FN	TN	TN	–	–	–	thyr ad; thyr ac
247	CVS, calcium antagonist.	FN	TN	TN	li; hrt	–	–	ut polyp; oral SCC
252	CVS, imidazoline agonist	FN	TN	TN	–	–	–	adr tu
272	CVS, Na-channel block	FN	TN	TN	–	li	–	thyr ad; tes ad; adr bpha; adr bpha
209	CVS, PDE3 inhibitor	FN	TN	TN	li; kid	–	–	adr bpha
232	CVS, remaining, D_1_/α agonist	FN	TN^a^	TN	adr; kid	–	–	pan ad
216	CVS, remaining, imidazole, PDE-inh	FN	TN^a^	TN	–	–	–	adr bpha
225	CVS, remaining, quinolone vasodilator	FN	TN^a^	TN	li; thyr; adr; spl; pros; tes	–	–	adr bpha
198	CVS, remaining, renin inhibitor	FN	TN^a^	TN	–	col	–	col ad; col ac
212	GI, 5HT4 agonist	FN	TN	TN	–	–	–	tes tu; pit ad
269	GI, 5HT4 agonist	FN	TN	TN	–	–	–	thyr ad; mam fad; pan ad; adr bpha; li ad; pit ad
210	GI, histamine H_2_ antagonist	FN	TN	TN	li	–	–	tes ad
275	GI, histamine H_2_ antagonist	FN	TN	TN	–	–	–	skin fibr
238	GI, remaining, sugar alcohol	FN	TN^a^	TN	–	li	–	tes tu
194	MB, antidiabetic, α-glucosidase inhibitor	FN	TN	TN	–	–	–	tes ad; kid ad; kid ac
195	RS, histamine H_1_ antagonist	FN	TN	TN	li; kid	–	–	adr bpha
207	RS, histamine H_1_ antagonist	FN	TN	TN	–	li	–	thyr ad; pit ac; li ac
264	RS, remaining, methylxanthine-derivate	FN	TN	TN	li	–	–	tes tu; mam fad
268	UB, anticholinergic	FN	TN	TN	–	li	–	ut polyp; kid pap
283	UB, anticholinergic	FN	TN	TN	–	–	–	kid sar
287	UB, anticholinergic	FN	TN	TN	–	–	–	skin sar
196	ZZ, remaining, retinoid, topical, keratinocyte	FN	TN^a^	TN	pit; adr	–	–	adr bpha; thyr ad
274	CNS, DA_2_ agonist	FN	TP	TP	adr	li	–	tes ad; skin fibr
245	CNS, DA_2_ agonist	FN	TP	TP	–	–	–	tes ad; tes ca
265	CNS, DA_2_ agonist	FN	TP	TP	–	–	–	pit ad; ut ac
270	CNS, DA_2_ agonist	FN	TP	TP	–	–	–	tes ad
273	CNS, DA_2_ antagonist	FN	TP	TP	–	–	–	islet ad; mam ac; pit ad
213	CNS, remaining, carbonic anhydrase inhibitor	FN	TP^a^	TP	–	–	–	UGT pap
278	CVS, α_1_ antagonist	FN	TP	TP	br; li; kid; hrt	–	–	adr bpha; mam ac
259	GI, proton pump inhibitor	FN	TP	TP	–	stom	–	stom tu; stom SCC; li ad
215	HM, dual 5-reductase inhibitor	FN	TP	TP	–	–	–	tes ad
224	HM, dual 5-reductase inhibitor	FN	TP	TP	–	–	–	thyr ad
221	HM, estrogen agonist	FN	TP	TP	–	–	–	pit ad
281	HM, estrogen agonist	FN	TP	TP	–	–	–	li ad; mam ca
254	HM, GnRH agonist	FN	TP	TP	–	–	–	adr bpha; adr mpha; islet ad; tes ad; pit ad; pit ca
286	HM, GnRH agonist	FN	TP	TP	–	–	–	pit ad; pit ca
257	HM, progestogen–estrogen contraceptive	FN	TP	TP	adr; li	–	–	pit ad; mam ad; mam ac
214	HM, progesterone antagonist, birth cont	FN	TP	TP	li	–	–	li ad; ut ac; mam ac
241	HM, remaining, aromatase inhibitor	FN	TP	TP	–	li	–	ova gca; UGT pap
201	HM, selective estrogen modulator	FN	TP	TP	–	–	–	kid ad; kid ac; ova ad
202	MB, fibrate	FN	TP	TP	–	–	–	tes tu; adr bpha; li ac
211	MB, fibrate	FN	TP	TP	li; kid; hrt; adr; tes	–	–	pan ad; stom tu; li ad; li ac
267	MB, HMG-CoA reductase inhibitor	FN	TP	TP	–	–	–	thyr ad; li ac
258	MB, remaining, inhib. growth hormone	FN	TP^a^	TP	–	–	–	sk sar; ut ac
228	RS, β_2_ agonist	FN	TP	TP	–	pan	–	thyr ad; thyr ac; ova leio; mam ac
280	RS, β_2_ agonist	FN	TP	TP	–	–	–	ova leio
288	RS, β_2_ agonist	FN	TP	TP	lu; hrt	hrt	–	ova leio; pit ad; pit ac
199	RS, β_2_ agonist	FN	TP	TP	li	–	–	thyr ad
227	RS, corticosteroid	FN	TP	TP	–	–	–	islet tu; adr bpha; skin sar
158	AB, remaining, bactericidal	FP	NT	FP	li; spl; kid; thyr	–	stom; ut; stom	–
157	CNS, SSRI	FP	TN	TN	–	li	li	–
159	CVS, α_1_ agonist	FP	TN	TN	–	–	mam	–
145	CVS, α_2_ agonist	FP	TN	TN	–	–	thy	–
149	CVS, α_2_ agonist	FP	TN	TN	–	–	islet	–
156	CVS, angiotensin II antagonist	FP	TN	TN	–	–	kid	–
162	CVS, angiotensin II antagonist	FP	TN	TN	–	kid	kid	–
147	CVS, β antagonist	FP	TN	TN	–	adr	thyr	–
148	CVS, β antagonist/α_1_ blocker	FP	TN	TN	li	–	li	–
151	MB, antidiabetic, remaining, SGLT-2 inhibitor	FP	TN^a^	TN	–	kid	kid	–
160	MB, remaining, 3 β-hydroxysteroid dehydrogenase	FP	TN^a^	TN	–	adr	adr	–
154	RS, histamine H1 antagonist	FP	TN	TN	–	–	mam	–
155	RS, histamine H1 antagonist	FP	TN	TN	li	li	pan	–
153	UB, remaining xanthine oxidase inhibitor	FP	TN^a^	TN	–	–	thyr	–
144	CVS, α_1_ antagonist	FP	TP	TP	–	–	mam	–
161	CVS, α_1_ antagonist and 5-HT_1A_	FP	TN	TP	–	–	bm	–
150	IS, immunosuppressive	FP	TP	TP	–	–	ln	–
152	IS, immunosuppressive, mTOR inhibitor	FP	TP	TP	–	stom; thyr	stom	–
146	MB, HMG-CoA reductase inhibitor	FP	TP	TP	–	–	li	–
2	AF, remaining, benzimidazole	TN	NT	TN	–	–	–	–
108	AI, COX2 inhibitor	TN	TN	TN	–	–	–	–
72	AI, COX2 inhibitor	TN	TN	TN	–	–	–	–
44	AI, NSAID	TN	TN	TN	kid	–	–	–
45	AI, NSAID	TN	TN	TN	–	–	–	–
50	AI, NSAID	TN	TN	TN	kid; spl	–	–	–
64	AI, NSAID	TN	TN	TN	–	–	–	–
74	AI, NSAID	TN	TN	TN	–	–	–	–
83	AI, NSAID	TN	TN	TN	li; kid	–	–	–
91	AI, NSAID	TN	TN	TN	hrt; adr; kid	–	–	–
124	AI, NSAID	TN	TN	TN	–	–	–	–
129	AI, NSAID	TN	TN	TN	–	–	–	–
71	AI, NSAID,	TN	TN	TN	–	–	–	–
7	AI, remaining	TN	TN^a^	TN	li	–	–	–
122	AI, remaining, cytokine modulator	TN	TN^a^	TN	–	–	–	–
73	AM, remaining, antimalarial	TN	NT	TN	–	–	–	–
97	AM, remaining, antiparasite	TN	NT	TN	–	–	–	–
123	AV,	TN	NT	TN	–	–	–	–
135	AV, herpes genitalis	TN	NT	TN	–	–	–	–
60	AV, immunostimulant	TN	NT	TN	li; kid; adr	–	–	–
104	AV, nucleoside inhibitor	TN	NT	TN	–	–	–	–
18	AV, protease inhibitor	TN	NT	TN	–	–	–	–
55	AV, viral DNA polymerase inhibitor	TN	NT	TN	–	–	–	–
3	BM, bisphosphonate	TN	TN	TN	–	–	–	–
33	BM, bisphosphonate	TN	TN	TN	–	–	–	–
87	BM, bisphosphonate,	TN	TN	TN	thyr; parath	bo	–	–
28	BM, remaining, calcium-mimetic	TN	TN^a^	TN	–	–	–	–
4	CNS, 5-HT_1b/d_ agonist	TN	TN	TN	–	thyr; li	–	–
107	CNS, 5-HT_1b/d_ agonist,	TN	TN	TN	–	–	–	–
95	CNS, 5-HT_3_ antagonist	TN	TN	TN	–	–	–	–
24	CNS, antiepileptic, Na-channel blocker	TN	TN	TN	–	li	–	–
49	CNS, antiepileptic, Na-channel blocker	TN	TN	TN	–	–	–	–
65	CNS, antiepileptic, Na-channel blocker	TN	TN	TN	–	li	–	–
66	CNS, antiepileptic, Na-channel blocker	TN	TN	TN	–	li	–	–
5	CNS, benzodiazepine	TN	TN	TN	–	–	–	–
142	CNS, benzodiazepine-like hypnotic	TN	TN	TN	–	–	–	–
143	CNS, benzodiazepine-like hypnotic	TN	TN	TN	spl; li; kid; tes; hrt; pit	li	–	–
84	CNS, opioid, μ-agonist	TN	TN	TN	–	–	–	–
132	CNS, opioid, μ-agonist, anticholinergic	TN	TN	TN	–	–	–	–
85	CNS, opioid, μ-antagonist	TN	TN	TN	–	–	–	–
86	CNS, opioid, μ-antagonist	TN	TN	TN	–	–	–	–
75	CNS, opioid, remaining, κ agonist	TN	TN^a^	TN	–	–	–	–
22	CNS, remaining 5-HT, 5-HT_1_-agonist	TN	TN^a^	TN	–	–	–	–
56	CNS, remaining, acetylcholinesterase inhib	TN	TN^a^	TN	–	sgl	–	–
96	CNS, remaining, AMPA glutamate antagonist	TN	TN^a^	TN	–	–	–	–
106	CNS, remaining, cannabinoid antagonist	TN	TN^a^	TN	–	–	–	–
20	CNS, remaining, DA-NA uptake inhibitor	TN	TN^a^	TN	li; adr; thyr	li	–	–
118	CNS, remaining, GABA-enhancer	TN	TN^a^	TN	–	–	–	–
138	CNS, remaining, GABA-metab. inhib.	TN	TN^a^	TN	–	–	–	–
81	CNS, remaining, MAO-A inhibitor	TN	TN^a^	TN	lu; kid; thyr; tes; ova	–	–	–
102	CNS, remaining, MAO-B inhibitor	TN	TN^a^	TN	–	li	–	–
136	CNS, remaining, nicotine agonist	TN	TN^a^	TN	–	–	–	–
137	CNS, SNRI	TN	TN	TN	–	–	–	–
103	CNS, SNRI	TN	TN	TN	–	–	–	–
29	CNS, SSRI	TN	TN	TN	–	–	–	–
54	CNS, SSRI	TN	TN	TN	–	–	–	–
112	CNS, SSRI	TN	TN	TN	kid	li	–	–
88	CNS, SSRI, 5-HT antagonist	TN	TN	TN	–	–	–	–
15	CVS, ACE inhibitor	TN	TN	TN	–	–	–	–
69	CVS, ACE inhibitor	TN	TN	TN	–	–	–	–
117	CVS, ACE inhibitor	TN	TN	TN	kid	kid	–	–
13	CVS, angiotensin II antagonist	TN	TN	TN	–	–	–	–
40	CVS, angiotensin II antagonist	TN	TN	TN	–	–	–	–
23	CVS, angiotensin II antagonist	TN	TN	TN	–	kid	–	–
10	CVS, anticoagulant	TN	TN	TN	–	–	–	–
14	CVS, β antagonist	TN	TN	TN	–	–	–	–
16	CVS, β antagonist	TN	TN	TN	–	–	–	–
17	CVS, β antagonist	TN	TN	TN	hrt; li	–	–	–
25	CVS, β antagonist	TN	TN	TN	pit; lu; hrt; spl; kid; adr; tes; ova; br	–	–	–
26	CVS, β antagonist	TN	TN	TN	–	–	–	–
126	CVS, β antagonist	TN	TN	TN	–	–	–	–
127	CVS, β antagonist	TN	TN	TN	–	–	–	–
9	CVS, calcium antagonist	TN	TN	TN	hrt; kid	adr	–	–
90	CVS, calcium antagonist	TN	TN	TN	spl; kid; ova; hrt; li; adr; br	–	–	–
92	CVS, calcium antagonist	TN	TN	TN	–	–	–	–
93	CVS, calcium antagonist	TN	TN	TN	–	–	–	–
38	CVS, class 1C antiarrhythmic	TN	TN	TN	thyr; li	–	–	–
53	CVS, class 1C antiarrhythmic	TN	TN	TN	hrt; li	–	–	–
6	CVS, endothelin antagonist	TN	TN	TN	–	li; int; adr; mam	nose; bm	–
115	CVS, endothelin antagonist	TN	TN	TN	–	–	–	–
105	CVS, imidazoline agonist	TN	TN	TN	adr; tes	–	–	–
98	CVS, loop diuretic	TN	NC	TN	–	–	–	–
100	CVS, Na-channel block	TN	TN	TN	–	–	–	–
101	CVS, Na-channel block	TN	TN	TN	–	li	–	–
77	CVS, PDE3 inhibitor	TN	TN	TN	adr	–	–	–
99	CVS, platelet aggregation inhibitor	TN	NC	TN	–	li; thyr	–	–
63	CVS, remaining, 5-HT_2_ antagonist	TN	TN^a^	TN	spl; li; kid; hrt; pan; br; thy; adr	–	–	–
141	CVS, remaining, β_1_ partial agonist	TN	TN^a^	TN	–	–	–	–
36	CVS, remaining, hemostatic	TN	TN^a^	TN	–	–	–	–
89	CVS, remaining, Nitr/K^+^ ATP agonist	TN	TN^a^	TN	–	–	–	–
113	CVS, remaining, PDE_5_ inhibitor	TN	TN^a^	TN	–	li; thyr	–	–
78	CVS, remaining, vasodilator	TN	TN^a^	TN	–	hrt	–	–
110	CVS, vasopressin-2 agonist	TN	TN	TN	–	–	–	–
131	CVS, vasopressin-2 agonist	TN	TN	TN	–	–	–	–
121	GI, 5-HT_4_ agonist	TN	TN	TN	–	–	–	–
48	GI, histamine H_2_ antagonist	TN	TN	TN	br; hrt; kid; tes; li; ova	–	–	–
94	GI, histamine H_2_ antagonist	TN	TN	TN	li; kid	–	–	–
119	GI, remaining, anti-osteoporose agent	TN	TN^a^	TN	–	–	–	–
32	GI, remaining, Fe-chelator	TN	TN^a^	TN	–	–	–	–
70	GI, remaining, opioid, μ-agonist	TN	TN^a^	TN	–	–	–	–
30	GI, remaining, phosphate binder	TN	TN^a^	TN	–	–	–	–
80	GI, remaining, synthetisch prostaglandin	TN	TN^a^	TN	adr; li	–	–	–
8	IS, remaining	TN	NC	TN	–	–	–	–
76	MB, antidiabetic, α-glucosidase inhib.	TN	TN	TN	–	–	–	–
68	MB, antidiabetic, DPP_4_ inhibitor	TN	TN	TN	–	thyr; li	–	–
111	MB, antidiabetic, DPP_4_ inhibitor	TN	TN	TN	–	–	–	–
114	MB, antidiabetic, DPP_4_ inhibitor	TN	TN	TN	–	–	–	–
139	MB, antidiabetic, DPP_4_ inhibitor	TN	TN	TN	–	–	–	–
58	MB, antidiabetic, remaining, SU derivative	TN	TN^a^	TN	–	–	–	–
130	MB, remaining, aldose reductase inhibitor	TN	TN^a^	TN	–	–	–	–
43	MB, remaining, hypertriglyceridemia	TN	TN^a^	TN	–	–	–	–
57	MB, remaining, lipid replacement	TN	TN^a^	TN	–	–	–	–
1	MB, remaining, nicotinic acid derived	TN	TN^a^	TN	–	–	–	–
61	RS, anticholinergic	TN	TN	TN	–	–	–	–
128	RS, anticholinergic	TN	TN	TN	–	–	–	–
11	RS, histamine H_1_ antagonist	TN	TN	TN	–	–	–	–
12	RS, histamine H_1_ antagonist	TN	TN	TN	li; lu; hrt; kid; tes	li	–	–
67	RS, histamine H_1_ antagonist	TN	TN	TN	–	li	–	–
82	RS, remaining, leukotriene receptor antagonist	TN	TN	TN	–	–	–	–
116	RS, remaining, mast cell stabilizer	TN	TN	TN	–	–	–	–
51	UB, anticholinergic	TN	TN	TN	–	–	–	–
125	UB, anticholinergic and calcium antagonist	TN	TN	TN	thyr; adr; ova; li	–	–	–
79	UB, remaining, oral β_3_ agonist	TN	TN^a^	TN	–	li	–	–
62	ZZ, remaining, CFTR potentiator	TN	TN^a^	TN	–	–	–	–
39	ZZ, remaining, prostaglandin E2	TN	TN^a^	TN	–	–	–	–
109	ZZ, remaining, protein kinase C-beta inhibitor	TN	TN^a^	TN	–	–	–	–
59	CNS, DA2-antagonist/5-HT antagonist	TN	TP	TP	–	–	–	–
19	CVS, α_1_ antagonist	TN	TP	TP	kid; br; tes	–	–	–
34	CVS, α_1_ antagonist	TN	TP	TP	–	–	–	–
133	CVS, α_1_ antagonist	TN	TP	TP	–	–	–	–
41	GI, proton pump inhibitor	TN	TP	TP	–	–	–	–
21	HM, GnRH agonist	TN	TP	TP	–	–	–	–
42	HM, progestogen–estrogen contraceptive	TN	TP	TP	pit; thyr	–	–	–
120	IS, immunosuppressive	TN	TP	TP	–	–	–	–
140	IS, immunosuppressive	TN	TP	TP	–	–	–	–
47	IS, immunosuppressive, mTOR inhibitor	TN	TP	TP	–	thyr	–	–
52	IS, immunosuppressive, S1P antagonist	TN	TP	TP	–	–	–	–
134	MB, antidiabetic, remaining, PPAR-γ	TN	TP^a^	TP	hrt; li	li	–	–
46	MB, fibrate	TN	TP	TP	–	–	–	–
27	MB, HMG-CoA reductase inhibitor	TN	TP	TP	–	–	–	–
35	CNS, SSRI	TN	TN	TN	–	–	–	–
37	CVS, ACE inhibitor	TN	TN	TN	–	–	–	–
31	UB, anticholinergic	TN	TN	TN	–	–	–	–
184	RS, remaining, antifibrotic	TP	NC	TN	–	adr	adr	li ad; ut ac
164	AI, NSAID	TP	TN	TN	–	–	kid; UGT	adr bpha
181	CNS, 5-HT_1b/d_ agonist	TP	TN	TN	kid	–	epi; tes	thyr ad; pit ad; thy bthym
176	CNS, 5-HT_3_ antagonist	TP	TN	TN	–	–	–	li ad; li ac
183	CNS, antiepileptic, Na-channel blocker	TP	TN	TN	kid; adr	li	kid	li ac
174	CVS, ACE inhibitor	TP	TN	TN	thyr	–	kid	pit ad; br ac; mes lip; pit ac
186	CVS, ACE inhibitor	TP	TN	TN	–	kid	kid	ln bhaem
167	CVS, α_2_ agonist, ocular	TP	TN	TN	–	int	int	pan ac; thyr ad; mam ad
165	CVS, calcium antagonist	TP	TN	TN	li	li	ln; thyr	thyr ad
172	CVS, calcium antagonist	TP	TN	TN	–	–	col	mam fad; adr bpha; tes ad; pit ad; mam ac; pit ca
182	AF, conazole derivative	TP	NT	TP	li; kid; spl; br; ova; thyr	–	thyr	tes tu; br astr; skin mel; mam ac
171	AV, guanosine analog	TP	NT	TP	–	pit	tes	mam ac; skin sar
189	AV, protease inhibitor	TP	NT	TP	–	thyr	li; kid	adr bpha
185	CNS, 5-HT_2_ antagonist	TP	NC	TP	li	thyr; mam	mam	thyr ad; mam ac
163	CNS, DA_2_-antagonist, benzamide	TP	TP	TP	–	–	mam	pan ad; pan ac; adr bpha; mam ca; pit ca
188	CNS, DA_2_-antagonist, DA3 antagonist	TP	TP	TP	li	–	lu	mam ca
177	CNS, remaining, electron transporter	TP	TP^a^	TP	–	–	stom	SCC and basal ca
192	CVS, α_1_ antagonist	TP	TP	TP	–	li; vag	li; mam	thyr ad; thyr ac
193	CVS, α_1_ antagonist	TP	TP	TP	–	–	mam	mam ad; hsyst leu
169	CVS, remaining, hydrazinophthalazine	TP	TP^a^	TP	–	pit	thyr	thyr ad; thyr ac
178	GI, proton pump inhibitor	TP	TP	TP	li; li; lu; stom	li; stom; stom	stom	tes ad; tes ad
187	GI, proton pump inhibitor	TP	TP	TP	li; kid; stom; thyr; hrt; spl	li; stom; thyr	stom	adr bpha; tes ad; stom SCP; stom SCC; hsyst leu; pit ad
175	HM, GnRH agonist	TP	TP	TP	–	–	tes	pit ad
180	HM, GnRH agonist	TP	TP	TP	br	pit	pit	pit ad
166	HM, remaining, antiandrogen	TP	TP	TP	tes; adr	li; ova; adr; thyr	tes; ova	te ad; thyr ad; ut ac
179	HM, selective estrogen modulator	TP	TP	TP	–	–	ova	kid ac; ova ad
190	MB, HMG-CoA reductase inhibitor	TP	TP	TP	–	li	li; stom	ut polyp
173	MB, HMG-CoA reductase inhibitor	TP	TP	TP	thyr	–	stom	stom SCP; thyr ac; thyr ad
191	RS, β_2_ agonist	TP	TP	TP	–	–	nose	ova leio; pit ad
168	RS, corticosteroid	TP	TP	TP	–	–	mam	mam fad; li ac; br astr; li ad
170	RS, corticosteroid	TP	TP	TP	many; tes; br; hrt; kid; pit; li	li	pan; ln	pan ad; pan ac; bo most; li ad; li ac; li ac; mam ad; mam ac

*^a^Decision on category is based on this single case*.

### Step 1: Categorization Based on Histopathology

We have categorized all compounds on the basis of histopathology findings in chronic studies and their relation to tumor findings in the 2-year studies, similar, but not identical, to the criteria in the work of Sistare et al. (16). The following four categories were identified:

1. Compounds that were negative for histopathological findings considered to be putative preneoplastic in the subchronic study and in the carcinogenicity study were considered true negatives (TNs). Compounds inducing hypertrophy only in subchronic studies were scored as negative.
2. Compounds that were positive for histopathology findings considered to be putative preneoplastic in the subchronic study, but negative for carcinogenicity, were classified as false positives (FPs).
3. Compounds that were positive for both the presence of treatment-related putative preneoplastic histopathological lesions in the subchronic study and the presence of treatment-related benign and/or malignant tumors in the carcinogenicity study were considered true positives (TPs).
4. Compounds that were negative for histopathology findings in the subchronic study, but positive for carcinogenicity, were considered false negatives (FNs).

### Step 2: Categorization Based on Pharmacology

For each pharmacological class, we have listed all compounds and the outcomes of the subchronic study and the carcinogenicity study. We have counted the number of compounds in a pharmacological class and the number of compounds inducing carcinogenicity.

- A class of compounds was called positive, when 75% of the compounds were associated with tumor induction. These classes are listed in Table 3.
- A class of compounds was called negative, when 75% of the compounds were not associated with tumor induction. These classes are listed in Table 4.
- A class of compounds was called with mixed outcome, when more than 25%, but less than 75%, of the compounds were associated with tumor induction. These classes are listed in Table 5.

After considering the mode of action (also based on publicly available literature) leading to induction of tumors, we made an evaluation of the probability that the pharmacology would be the main mode of action causing the carcinogenicity. We have discussed this and added to the tables as the proposed final categorization.

## Results

A total of 366 pharmaceuticals have been evaluated in the present study, of which 289 met the criteria described in Section “Materials and Methods” for defining valid pairs of rat subchronic (3 and/or 6-month) and 2-year carcinogenicity studies.

### Genotoxicity Evaluation

In our dataset, 21 compounds were assessed as positive or inconclusive with respect to genotoxicity. To give a detailed description is not relevant, since this assessment is conducted initially during the assessment for marketing authorization. After discussions with the sponsor, and before making a decision about the authorization, it was agreed that the genotoxicity findings do not influence the risk for patients. We have therefore decided not to give any weight to these data. None of those compounds is intended to be given to patients with a life-threatening disease, in which case a serious benefit would be judged to outweigh the genotoxicity risk as specified in ICH S1A (2).

### Histopathological Classification

#### True Negative Compounds

One hundred forty-three (50% of the total) pharmaceuticals that did not induce putative preneoplastic lesions in the subchronic (3- and/or 6-month) study also did not cause treatment-related tumors in the carcinogenicity study (Table 1, third column). Ninety-nine of these 143 compounds (69%) did neither exhibit any effect on organ weight nor induce cellular hypertrophy in any organ (Table 1).

Ninety-one of the 143 TN compounds (31% of the total) demonstrated a greater weight of one or more organs, such as liver (*n* = 16); kidneys (*n* = 17); heart (*n* = 11); adrenals (*n* = 10); spleen, ovaries, and brain (*n* = 5); testes (*n* = 7); thyroid (*n* = 6); pituitary and lungs (*n* = 3), or incidentally (*n* = 1) other organs, either or not in combination with cellular hypertrophy (2 substances in liver and 1 substance in kidneys). Twenty-six pharmaceuticals showed cellular hypertrophy in the liver (*n* = 13), thyroid (*n* = 5), adrenals (*n* = 2), and incidentally in the kidneys, mammary glands, bone, salivary glands, or intestines.

Cellular hypertrophy was mainly observed in the liver (*n* = 17), in four cases accompanied by greater liver weight, but not accompanied by the development of benign or malignant hepatocellular tumors.

#### False Negative Compounds

Ninety-six (33% of the total) substances did not exhibit putative preneoplastic lesions in the subchronic study, whereas treatment-related benign and/or malignant tumors developed in the carcinogenicity study.

Fifty-two of these 96 compounds (54%) induced benign tumors in a single organ (39) or in multiple organs (13), mainly testes (Leydig cell adenomas), adrenals (pheochromocytomas), pancreas (acinar cell adenomas), thyroid (follicular cell adenomas), pituitary (pars distalis adenomas), and liver (hepatocellular adenomas). Incidental benign tumors were seen in mammary gland, skin, ovaries, urogenital tract, and spleen.

Thirty-five of the 96 compounds (36%) induced benign and malignant tumors in the same organ, mainly liver, thyroid, testes, mammary gland, and pancreas (9%), or in multiple organs (27%).

Nine of the 96 compounds (9%) induced malignant tumors only in a single or multiple organs. These malignant tumors comprised skin sarcomas; adenocarcinomas of the uterus, a localized lymphoma; adenocarcinomas in the kidneys; and leukemia.

Seventy-seven of the 96 compounds (80%) caused tumors that frequently occur spontaneously in rats of the age examined and most of them are not considered relevant for humans.

#### False Positive Compounds

Of the 50 compounds that induced putative preneoplastic (hyperplastic) histopathological lesions in the subchronic study, 19 of these (38%) failed to induce treatment-related tumors in the carcinogenicity study.

For these FP compounds, the site of histopathological evidence (cellular hyperplasia) of risk for rat neoplasia was the mammary glands, kidneys, and liver (three compounds each); thyroid and pancreas (two compounds each); and adrenals, stomach, uterus, lymph nodes, thymus, and bone marrow (one compound each).

#### True Positive Compounds

Thirty-one substances (11% of the total number) induced putative preneoplastic (hyperplastic) histopathological changes in the subchronic study and treatment-related neoplasms in the carcinogenicity study. In only 13 out of these 31 compounds (42%), the hyperplastic lesion and the tumor developed in the same organ, whereas for the other 18 compounds (58%), the hyperplastic lesions observed in the subchronic study did not develop in the same organ as the tumor in the carcinogenicity study.

Four compounds caused mammary gland hyperplasia in the subchronic study and mammary gland adenomas or carcinomas in the carcinogenicity study. One compound caused mammary gland hyperplasia without the development of mammary gland tumors. This compound induced follicular cell adenomas and carcinomas in the 24-month study but no follicular hyperplasia in the thyroid in the subchronic study. Six substances induced mammary gland (fibro)adenomas and/or adenocarcinomas in the mammary gland without mammary gland hyperplasia in the subchronic study.

### Pharmacological Analysis of the Carcinogenic Response

The 289 human pharmaceuticals in the dataset were distributed over therapeutic areas as indicated above (Table 1, fourth column). Most of the therapeutic areas are divided over several pharmacological classes, and all compounds are distributed through these classes. However, the compounds are anonymized (see Table 1) due to intellectual-property reasons. A similar approach was followed as for the previous paper on the PhRMA–FDA–JPMA dataset (PFJ dataset) (26).

#### CNS Drugs

##### DA_2_ Agonists [1], Refers to the Class in Tables 3–5

All four (*245, 265, 270*, and *274*) were found to induce tumors in the sexual organs. For three compounds (*245, 270*, and *274*), Leydig cell adenomas were observed, while for the fourth compound (*265*), uterine carcinomas have been described. For one compound (*274*), skin fibromas were observed too, while for another (*265*), a decrease in spontaneous pituitary adenomas was seen. The dopaminergic DA_2_ agonists are associated with an increase of luteinizing hormone, and the fact that all four compounds induced either testis tumors or uterus tumors confirms the association. In the PFJ dataset (26), only two compounds were included, with one showing the same tumor profile. Based upon the literature (30), we could find support for a pharmacodynamic relationship and so we categorized this class as TP.

##### DA_2_ Antagonists [2]

Three out of four (*59, 163, 188*, and *273*) showed mammary gland adenocarcinomas (two TP and one FN). For one compound, this was the only type of tumor observed (*188*). A second compound (*273*) induced in addition pancreatic acinar cell adenomas and pituitary adenomas. The third compound (*163*) induced adrenal pheochromocytomas, in addition to mammary gland carcinomas. In a 3-month study, mammary hyperplasia was observed for this compound making it a TP. The fourth compound (*59*) was a TN. It is well known that administration of dopaminergic DA_2_ antagonists is associated with an increase in prolactin, resulting in mammary adenocarcinoma in rodents. Based on this pharmacological effect, we categorized this class as TP.

##### 5HT_1b/d_ Agonists [43]

Four triptanes (*4, 107, 181*, and *277*) showed a rather mixed response. Two compounds (*4* and *107*) did not induce either tumors or hyperplastic responses after 6 months (TN). Two other compounds induced a variety of tumors, one (*277*) with benign pheochromocytomas and Leydig cell adenomas (FN), while with the other (*181*), thyroid follicular cell adenomas and thymomas were observed. However, with the latter compound, Leydig cell hyperplasia was observed at 6 months (TP). Effects on thyroid and testis (Leydig cell hyperplasia and adenoma) are likely be related to liver enzyme induction. In both cases, these effects cannot be related to the direct pharmacodynamic action, which is in agreement with the absence of proliferative effects (31). A general category of TN is given to this class of compounds.

##### 5HT_3_ Antagonists [44]

5HT_3_ antagonists (*95* and *176*) have an inhibitory effect on the growth of HT29 cells (32). Two 5HT_3_ antagonists showed different responses, one (*95*) was TN, while the other (*176*) induced liver adenocarcinomas. As this is unrelated to its pharmacology, the class was categorized as TN.

##### Serotonin and Noradrenaline Reuptake Inhibitors (SNRI) [47]

Four SNRIs (*137, 203, 250*, and *276*) are included with two (*137* and *103*) as TN, while the other two compounds (*250* and *276*) as FNs showed thyroid adenomas or testicular adenomas, respectively. As these tumors were not associated with primary pharmacology, this class was categorized as TN.

###### μ-Opioid Antagonists [26]

Two are included (*85* and *86*), both TN, and we categorized the class in this way.

###### CNS, Na^+^ Channel Blockers [46]

From six antiepileptics sharing the property of being sodium channel blockers (*24, 49, 65, 66, 183*, and *239*), four are TNs, one compound (*183*) showed liver hyperplasia at 6 months and hepatocellular adenocarcinomas after 2 years (TP). The sixth compound (*239*) showed only adrenal pheochromocytomas after 2 years (FN), which are not relevant for humans. The class is categorized as TN.

###### Selective Serotonin Reuptake Inhibitors [27]

Seven compounds (*29, 35, 54, 112, 157, 262*, and *88*) are included. Five were TNs, with one of them (*112*) showing hepatic hypertrophy, but no hyperplasia, at 6 months. The sixth compound (*262*) induced lymphoreticulum cell tumors (FN), while the seventh (*157*) only showed liver hyperplasia (FP). There is no direct relation with pharmacology, and the relevance for humans is estimated to be negligible. Therefore, selective serotonin reuptake inhibitors (SSRIs) are categorized as TN.

###### μ-Opioid Agonists [42]

Of the three μ-opioid agonists, two (*84* and *132*) are TN, whereas one (*205*) compound showed testis tumors and leukemia (at a non-matching dose). As opiates are not associated with proliferative action, this group was categorized as TN.

###### Benzodiazepine(-Like) Compounds [45]

Three real benzodiazepines and two benzodiazepine-receptor agonists are in this class. Three (*5, 142*, and *143*) are TN. One benzodiazepine (*204*) showed treatment-related thymus lymphomas, thyroid follicular cell adenomas, and uterus schwannomas, especially at high dosages, while another (*248*) showed only thyroid follicular cell adenomas. The latter compound also induced an increase in thyroid weight at 6 months. As these effects were associated with induction of liver metabolism rather than attributed to the primary pharmacology, this class was categorized as TN.

###### 5HT_2_ Antagonists [16]

Two compounds (*185* and *220*) are included. Compound *185* induced mammary gland hyperplasia after 3–6 months, and mammary gland adenocarcinomas and thyroid follicular cell adenomas after 2 years (TP). Compound *220* induced only liver adenomas and no effects at 6 months. There are signals that 5HT_2_ antagonists induce an increase in prolactin, which might be responsible for the association with mammary gland tumors. This has also been discussed with the PFJ dataset (26). However, this relation between 5HT_2_-receptor blockade and prolactin is not without discussion, and in this case, compound 185 might have also anti-DA_2_ affinity. Because of this uncertainty with respect to the pharmacology, we did not apply a category based upon pharmacology for this class (NC).

###### Remaining 5HT Compounds

One compound (*22*), a 5HT_1_ agonist was TN. We maintained for the 5HT_1_ agonist the TN category.

###### Remaining Opioid Compounds

One κ-agonist (*75*) is a TN, which is what we categorized it too.

###### Remaining CNS Compounds

Seventeen compounds with a large variety of pharmacological targets remained. Nine TNs are a nicotine agonist (*136*), an acetylcholinesterase inhibitor (*56*), a GABA-enhancer (*118*), a GABA-metabolism inhibitor (*138*), a dopamine–noradrenaline reuptake inhibitor (*20*), a monoamine oxidase inhibitor A (MAO-A) (*81*), a MAO-B inhibitor (*102*), an AMPA glutamate antagonist (*96*), and a cannabinoid antagonist (*106*). Several FNs were also present. An α_2_δ agonist of the L-calcium channel (*230*) induced pancreatic acinar cell adenomas and acinar cell adenocarcinomas, and Leydig cell adenomas in males and uterine endometrial polyps in females (33). A direct pharmacological explanation could not be found in relation to this receptor (34). Therefore, we maintained a category FN. A melatonin receptor agonist (*197*) induced hepatocellular adenomas and carcinomas, and we gave a TN categorization because of the absence of an association with melatonin receptor stimulation. A COMT inhibitor (*217*) was associated with an increase in kidney tubular adenomas and carcinomas. We maintained category FN, as the kidney effect is likely to be an off-target effect (i.e., not related to pharmacology). An NMDA antagonist (*223*) induced Leydig cell tumors. However, NMDA are usually negative (26) and therefore were assigned a TN category. A nootropic drug (*261*) was associated with adrenal pheochromocytomas, but categorized as TN, as these tumors are not relevant to humans (see below). A tetracyclic antidepressant (*251*) induced liver adenomas and thyroid follicular cell adenomas based upon induction of metabolism, and also mammary gland tumors, and therefore, we categorized this compound as FN, as there was obvious pharmacological explanation for the latter tumor. A carbonic anhydrase inhibitor (*213*) induced urinary bladder papillomas. These compounds are known to be associated with crystallization in rat urinary bladder, and we applied a category TP for this compound. An electron transporter (*177*) is labeled as TP inducing forestomach hyperplasia in 6-month studies and forestomach squamous cell and basal cell carcinomas in the 2-year study.

Several CNS compounds were associated with adrenal pheochromocytoma, i.e., the DA_2_ antagonist *163*, the NA-channel blocker *23*9, and the nootropic drug *261*. Pheochromocytoma (a tumor developing from the chromaffin cells, which are the sites of synthesis and storage of catecholamines) is the most common neoplasia of the adrenal medulla in rodents. Pheochromocytomas are frequently found in a background of diffuse medullary hyperplasia. Compounds producing this feedback interference include lactose and sugar alcohols such as lactitol and Ca^2+^. High doses of low digestibility carbohydrates, such as mannitol, sorbitol, xylitol, and lactitol, have been reported to increase the absorption and urinary excretion of Ca^2+^ as well as the incidence of all types of proliferative lesions in the adrenal medulla. Hypercalcemia is known to increase catecholamine synthesis. Other compounds that might act *via* altered Ca^2+^ homeostasis and progressive nephrocalcinoses in aging rats include the retinoids. Vitamin D is the most potent *in vivo* stimulus, yet identified for chromaffin cell proliferation in the adrenal medulla. Vitamin D_3_ resulted in a fourfold to fivefold increase in bromodexoyuridine (BrdU) labeling (35) in the adrenal medulla (focal hyperplasia), leading to pheochromocytomas. In the PFJ dataset, we identified four vitamin D-analogs, all associated with adrenal pheochromocytoma (26).

#### Cardiovascular Drugs

##### ACE Inhibitors [33]

Eleven angiotensin-converting enzyme inhibitors showed a variety of classifications. Four (*15, 37, 69*, and *117*) are TN. Two (*174* and *186*) are TP based on kidney hyperplasia at 6 months, and for one compound (*174*), pituitary gland adenocarcinomas and mesentery lipomas at 2 years, while for the other compound (*186*), benign hemangiomas were observed in the lymph node. Five compounds (*208, 266, 271, 285*, and *233*) are FN with kidney adenomas in compound *271*, mammary fibroadenomas in compound *285*, Leydig cell tumors in compound *208*, thyroid follicular cell adenomas with compound *266*, and thyroid follicular cell adenomas and endometrial polyps with compound *233*. Van der Laan et al. (26) mentioned seven negative compounds. The kidney as the target organ of compound *271* suggests a pharmacological effect that might be related to kidney hyperplasia (juxtaglomerular hyperplasia), which was seen with compounds *174* and *186*, as well as with angiotensin II antagonists (26). Mammary gland fibroadenomas, as seen with compound *285*, are the most common spontaneous tumors in female rats in almost all the routinely used rat strains with incidences of up to 70% in carcinogenicity studies. Fibroadenomas do not progress to malignancy and are not considered to be relevant for humans, whereas mammary gland adenocarcinomas in rats may be more relevant (35, 36). It is important to consider that the windows of mammary gland susceptibility or mammary gland sensitivity are missed when exposure starts in adult nulliparous rodents as is routinely the case in bioassays with pharmaceuticals. The tumors induced by *208* and *266* are likely to be associated with induction of liver metabolic enzymes. The variety of tumors seen with compound *174* is complex. We have categorized the ACE inhibitors as TN in accordance with Van der Laan et al. (26) overruling all other categories for individual compounds.

##### Ca Antagonists [14]

Twelve Ca antagonists are included in this dataset. Four are TN (*9, 90, 92*, and *93*). Two (*165* and *172*) are TP, the first with lymph node hyperplasia at 6 months and thyroid follicular cell adenomas after 2 years, and the other showed colon hyperplasia of the muscularis mucosa at 6 months and after 2 years Leydig cell adenomas in the testis, mammary gland fibroadenomas, adrenal pheochromocytomas, and pituitary adenomas and carcinomas. Six others compounds (*200, 235, 237, 240, 256*, and *247*) are FN. Two compounds (*235* and *237*) induced Leydig cell tumors, one induced thyroid follicular cell adenomas (*256*), and one compound (*240*) was associated with pituitary gland adenomas and mammary gland fibroadenomas. Compound *200* induced uterine polyps, while compound *247* induced uterine polyps and oral mucosa squamous cell carcinomas. Calcium antagonists, especially dihydropyridines, are not associated with tumor induction (37). Eight out of 12 compounds in our dataset are associated with the induction of tumors. Two compounds (*165* and *256*) induced thyroid tumors only, and two (*235* and *237*) only induced testis tumors. Two compounds (*172* and *240*) induced mammary fibroadenomas and pituitary tumors. Compound *247* induced uterine polyps (as did compound *200*) and specifically gingival squamous cell carcinoma. The latter phenomenon is reported for mibefradil (38), as being due to the oral intake as diet mixture, and not directly related to the pharmacological effect. Van der Laan et al. (26) classified calcium antagonists as negative, in accordance with absence of induction of cancer in humans (39). We, therefore, categorized all Ca antagonists as TN, taking into account these considerations.

##### Angiotensin II Receptor Antagonists [21]

Five compounds (*13, 40, 156, 23*, and *162*) were included. Three compounds (*13, 23*, and *40*) are TN, and two (*156* and *162*) showed juxtoglomerular hyperplasia at 6 months (FP). The induction of juxtaglomerular hyperplasia does not predict further development to kidney tumors, which confirms the findings of Van der Laan et al. (26), with a slightly different sample set (only two compounds *overlap*). We categorized the class as TN.

##### Adrenergic α_1_ Antagonists [34]

Eight adrenergic α_1_ antagonists (*19, 34, 133, 144, 192, 193, 278*, and *161*) showed different target organs, with three TN (*19, 34*, and *133*). Three compounds showed mammary gland hyperplasia (*144, 192*, and *193*) and two (*193* and *278*) mammary gland tumors. Two compounds (*192* and *193*) are TP with mammary gland hyperplasia at 6 months and mammary gland adenomas and mononuclear cell leukemia after 2 years. Two (*144* and *161*) are FP, with mammary gland acinar hyperplasia at 6 months for one (*144*) and bone marrow hyperplasia at 6 months for the other (*161*). One FN (*278*) showed after 2 years mammary gland adenocarcinomas and adrenal phaechromocytomas. A direct pharmacological explanation for this connection to the mammary system is unknown at this time. In addition, a connection between α_1_ antagonism and mammary tumor formation is also unknown. A choice for the final classification TP is based on the effects on the mammalian gland, both after 6 months and 2 years for several compounds, despite the fact that we have no clear molecular mechanism. Further research is, therefore, important to study this possible association of α_1_ antagonism and mammary tumor formation.

##### Adrenergic α_1_ Agonists [35]

Two compounds are included; one (*249*) is FN with only Leydig cell adenomas (related to enhanced liver metabolism) at 2 years, while the other (*159*) was FP with mammary gland acinar hyperplasia at 6 months but no tumors at 2 years. TN was chosen for the final categorization.

##### Adrenergic α_2_ Agonists [36]

Three compounds (*145, 149*, and *167*) are included, of which one (*145*) is a TN. Compound *149* is FP with hyperplasia of the islets of Langerhans in the pancreas, while compound *167* is TP with small intestines hyperplasia at 6 months and pancreas acinar adenocarcinomas, thyroid follicular cell adenomas, and mammary gland adenomas after 2 years. Three adrenergic α_2_ agonists, therefore, showed variable effects. The islet cell hyperplasia seen with compound *149* might be related to the pancreatic adenocarcinoma seen with compound *167*. A pharmacological target in the pancreas for this class is well known to be inhibitory, e.g., inhibiting insulin secretion. Other compounds in this class have been mentioned in the literature as negative (23, 26, 40), and therefore, this group is categorized as TN.

##### Adrenergic β-Antagonists [25]

Thirteen β-blockers (*14, 16, 17, 25, 26, 126, 127, 147, 148, 203, 219, 243*, and *255*) are included, with seven categorized as TN. Two FP (*147* and *148*) showed thyroid follicular cell hyperplasia or hepatocellular hyperplasia but no tumors. Of the four FN, compound *201* showed hepatocellular hepatomas, compound *219* showed forestomach squamous cell papillomas, compound *243* showed pituitary gland tumors, and compound *255* showed spleen vascular neoplasia. The carcinogenicity potential of β-blockers has been a debate from their early existence, especially with respect to pronethalol (41).The tumors found were heterogeneous and therefore probably not related to pharmacology. Snyder and Green (23) also mentioned a low incidence of compounds associated with tumors for this class (2 out of 10). This class was therefore categorized as TN overruling the FN compounds.

##### Anticoagulants [37]

Two anticoagulants (*10* and *289*) showed slightly different outcomes. The tumor outcome of compound *289* was in fact not statistically relevant but was decided to be a safety signal. However, pancreatic acinar adenomas/carcinomas are usually not relevant for humans. Therefore, we applied a category TN.

##### Imidazoline Agonists [38]

Two compounds are included (*105* and *252*): one is TN, while the other is FN with adrenal phaechromocytomas and hind limb tumors after 2 years. The adrenal tumors seen with compound *252* might be reflected by a greater adrenal weight for compound *105*, although this might be speculative. The human relevance is low anyway. Therefore, it was decided to categorize these as TN.

##### CVS, Na-Channel Blockers [39]

Of the three Na-channel blockers used in cardiac treatment (*100, 101*, and *272*), two were TN, compound *227* showed thyroid and Leydig cell adenomas, as well as adrenal pheochromocytomas. As these tumors are more related to drug metabolism, and not pharmacology, we categorized this class as TN.

##### Loop Diuretics [16]

Three of the four (*98, 229, 253*, and *284*) compounds are FN with compound *284* causing kidney carcinomas, compound *229* causing thyroid follicular cell carcinomas and pituitary gland adenomas, and compound *253* causing uterus adenocarcinomas and Leydig cell adenomas. Compound 284 is a FN, and its target organ suggests a pharmacological profile for the carcinogenesis. The target organs of the other loop diuretics are probably not associated with their pharmacology. As this positive relationship was found for only one compound, we decided to leave the categorization for this class as undecided (NC: non-categorizable based on pharmacological target), with no change of the histopathological categories.

##### Class 1C Channel Blockers [22]

Two compounds (*38* and *53*), are included, both are TNs, and we also applied this categorization to these based on their pharmacology.

##### Endothelin Antagonists [23]

Two compounds (*6* and *115*) are included, both are TNs. No proliferative effects are reported for these compounds, and we categorized them as TN based on pharmacology.

##### Vasopressin-2 Agonists [24]

Two compounds (*110* and *131*) are included and both were TN. No proliferative effects are reported for these compounds, and we categorized them as TN based on pharmacology.

##### Platelet Aggregation Inhibitors [40]

Two compounds (*99* and *282*) are included. One (*99*) was TN. The other (*282*) showed a variety of tumors, such as thyroid follicular cell adenomas, adrenal pheochromocytomas, hepatocellular adenomas, and endocrine-related tumors such as uterine adenocarcinomas, ovarian adenomas, and mammary gland adenomas. Two platelet aggregation inhibitors had a different tumor response; the first was TN, whereas the other compound was FN because of a dopaminergic action as a secondary pharmacological effect (42). These compounds were not categorized related to pharmacology (NC).

##### Phosphodiesterase 3 Inhibitors [41]

One compound (*77*) was TN, while with the other (*209*), adrenal pheochromocytomas were observed. Therefore, we decided to categorize the class as TN.

##### Remaining CVS Compounds

Eleven compounds with a variety on pharmacological targets remained to be categorized. A 5HT_2_ antagonist (*63*) is TN, as is a β_1_-partial agonist (*141*). Five other TNs are a nitrate agonist (*89*), a phosphodiesterase-5 inhibitor (*113*), a sodium-channel inhibitor, a hemostatic compound (*36*), and a vasodilator (nitric oxide agonist) (*78*). All compounds were maintained in the TN category. A hydrazine (*169*) is TP with thyroid follicular cell hyperplasia after 6 months and thyroid follicular cell adenomas and carcinomas after 2 years. Because of the thyroid hyperplasia (without any liver signal) after 6 months, we maintained the TP category in this case, although a relation with pharmacology needs to be substantiated. Four compounds are FN, i.e., a DA_1_/α_1_ agonist (*232*) that induced pancreatic acinar cell adenoma, an imidazole PDE inhibitor (*216*) that was associated with adrenal pheochromocytomas, a quinolone vasodilator (*225*), and a renin inhibitor (*198*) that was associated with colon adenoma and carcinoma. However, the colon is unlikely to be a pharmacological target, as Kochi et al. (43) described an antagonistic effect for this relation. Therefore, we decided to give a TN category to this class.

#### Gastrointestinal System

##### Proton Pump Inhibitors [12]

Four compounds (*41, 178, 187*, and *259*) are included, with one TN. One TP (*187*) showed stomach hyperplasia after 3 months, and monocytic cell leukemia, adrenal pheochromocytomas, pituitary adenomas, Leydig cell adenomas, and squamous cell carcinomas in the forestomach. Another TP (*178*) showed stomach hyperplasia at 6 months and Leydig cell adenomas at 2 years. The third compound (*259*) was associated with hepatocellular adenomas and squamous cell carcinomas in the stomach. The fourth compound was TN. The stomach hyperplasia and squamous cell carcinoma are clearly related to the pharmacology of this class. Therefore, we decided that this class should be categorized as TP, overruling the TN case.

##### 5HT_4_ Agonists [18]

From the three compounds (*121, 269*, and *212*), one compound (*121*) is a TN, while the other two are FN with tumors in the 2-year studies. Compound *269* showed Leydig cell adenomas and pituitary adenomas, while compound *212* showed thyroid follicular cell adenomas, mammary gland fibroadenomas, pancreatic acinar cell adenomas, adrenal pheochromocytomas, hepatocellular adenomas, and pituitary adenomas. The GI 5HT_4_ agonists are likely to be associated with indirect metabolic effects on thyroid hormones (*269*) or testosterone (*212*). However, this can be debated for compound *269*, but a direct pharmacological explanation is not known. We categorized all these compounds, therefore, as TN.

##### Histamine H_2_ Antagonists [49]

From the four compounds, two (*48* and *94*) are TN. The other two (*210* and *275*) are FN, with *275* associated with an increased number of skin fibromas, and the compound *210* with Leydig cell adenomas. Histamine H_2_ antagonists are used as gastric acid secretion inhibitors. Inhibition of gastric secretion might be associated with long-term induction of gastric carcinoids as associated with their pharmacological action. None of the compounds in this dataset showed this effect. Therefore, these were categorized as TNs, although in the previous paper, the H_2_ antagonists belonged to the positive class.

##### Gastrointestinal, Remaining Compounds

Five compounds remained in this class, and four were categorized as TN, i.e., a synthetic prostaglandin (*80*), a phosphate binder (*30*), an imaging/anti-osteoporose agent (119), and a Fe-chelator (*32*). A sugar alcohol (compound *238*) is associated with Leydig cell adenomas (FN). As this is a rather unspecific effect, the compound was categorized as a TN.

#### Hormonal System

##### GnRH Agonist [3]

From the five compounds (*21, 175, 180, 254*, and *286*), one is TN (*21*) while two are TP (*175, 180*), one with pituitary hyperplasia (*180*) after 3 months and pituitary adenomas after 2 years, and the other showed Leydig cell hyperplasia after 6 months and pituitary adenomas after 2 years. Two compounds (*254* and *286*) are FN, with no hyperplastic effects after 6 months, but both associated with pituitary adenomas and carcinomas after 2 years. One of these compounds (*254*) showed in addition Leydig cell adenomas and pancreatic islet cell adenomas, and adrenal benign and malignant pheochromocytomas. The relation between pharmacology of GnRH agonists and the pituitary tumors observed for four compounds is clear, as the target organ is the pituitary (44). Only one compound (*21*) is TN, but we categorize all as TP based upon the similar pharmacology as the other members of the class.

##### Estrogen Agonist [4]

Two compounds (*221* and *281*) are included; one compound (*221*) with pituitary gland adenomas, and the other (*281*) with mammary gland carcinomas and hepatocellular adenomas. For the estrogen agonists, the identity of the target organ supports a pharmacological relationship. Estrogen agonists are important as potential human-relevant carcinogens based upon IARC evaluations (45), and we categorized this class, therefore, as TP.

##### Selective Estrogen Receptor Modulators [5]

Two compounds (179 and 201) have a similar tumor profile, i.e., kidney adenocarcinomas and ovary adenomas. Only one (179) showed ovary hyperplasia after 6 months making it a TP, while the other (201) was an FN. We categorized this class as TP.

##### Dual 5-Reductase Inhibitors [6]

Two compounds (215 and 224) are included, and these are FN. One compound induced Leydig cell adenomas, while the other induced only thyroid follicular cell adenomas. Because of their hormonal action, we categorized these compounds as TP, although the possibility that the tumors might be the consequence of just the induction of liver metabolism cannot be discounted.

##### Progestogen–Estrogen Combinations [7]

From the two combinations (*42* and *257*), one (*42*) is a TN and the other is a FN (*257*), with mammary gland adenocarcinomas and pituitary adenomas after 2 years. A pharmacological effect on the basis of estrogenic activity is considered to be likely, and therefore, we applied a TP category.

##### Hormones, Remaining Compounds

An antiandrogen (*166*) is a TP, with Leydig cell hyperplasia and ovary hyperplasia observed at a 3- or 6-month study. Leydig cell adenomas and thyroid follicular cell adenomas and uterus adenocarcinomas were seen after 2 years. An aromatase inhibitor (*241*) is FN, with urinary bladder papillomas and ovary benign stromal cell tumors after 2 years.

For all pharmacological classes associated with sexual hormones, we conclude that such a relationship is likely to exist between pharmacological action and the induction of tumors, such as for the dual 5-reductase inhibitors (*215* and *224*), and the progestogen-containing combinations (*42, 214*, and *257*). Therefore, we categorized all of these compounds as TP.

#### Immunological System

##### Immunosuppressives [29]

Six immunosuppressive compounds (*120, 140, 150, 47, 152*, and *52*) are all negative. Four compounds are TN and two FP. One of the FPs, compound *150*, showed lymph node hyperplasia at 6 months, and compound *152* showed stomach hyperplasia at 6 months. The fact that all compounds are negative is remarkable, as the immunosuppressive action is a well-known risk factor for the induction of cancer. Bugelski et al. (46) showed that around 50% of immunosuppressive compounds were associated with some type of cancer, probably based upon the spontaneous presence of oncogenic viruses. The absence of a carcinogenic effect for these immunosuppressive compounds could be explained by an absence of oncogenic viruses during these studies. It is known that immunosuppression is a real risk factor for human carcinogenicity. From that point of view, the ICH-S1 Expert Working Group indicated that compounds with an immunosuppressive risk could warrant a waiver in the future. Cyclosporin is also a class 1A (IARC) proven human carcinogen. Therefore, we decided to categorize the class of immunosuppressives as TP, despite the lack of tumors in these studies.

##### Immunomodulators [52]

Two compounds are included. Compound *8* is a TN, while compound *242* is a FN with pituitary adenomas noted at 2 years. Categorization of this class based on pharmacology remains uncertain. Therefore, we maintained the histopathological categorization for these compounds.

#### Metabolic System

##### Antidiabetics, α-Glycosidase Inhibitors [50]

Two compounds (*76, 194*) are included; one (*76*) is TN. The other has been studied in relation to glucose inclusion in the diet and as a pair-fed study. The study with glucose resulted in Leydig cell adenomas after 2 years, whereas without glucose, kidney tumors were observed. A final pair-fed study did not reveal any treatment-related increase in tumors. As the target of α-glycosidase inhibitors is rather the intestine than the kidney, we concluded that this should be considered an off-target effect. We categorized this class, therefore, as TN.

##### Dipeptidyl Peptidase Inhibitors (DPP4 Inhibitors) [28]

Four DPP4 inhibitors (*68, 111, 114*, and *139*) were all TN. Apparently, the inhibition of the breakdown of GLP-1 is only low and does not lead to the induction of thyroid C-cell tumors, as is known from GLP-1 agonists such as liraglutide (47). We categorized this class as TN.

##### PPAR-α-Agonists (Fibrates) [8]

Three compounds are included (*46, 202*, and *211*). One is TN (*46*). Two are FN, with both compounds inducing hepatocellular carcinomas, accompanied with pancreas acinar cell adenomas and forestomach squamous cell carcinomas for compound *211*. Compounds *46* and *202* showed Leydig cell adenomas and adrenal pheochromocytomas. Fibrates are known to target the liver, but the pancreas can also be listed as a target organ based on their class properties related to peroxisome proliferation (48). Because of the pharmacological profile, the histopathological categorization TN and FN, respectively, was considered to be overruled by TP for this class.

##### HMG-CoA Reductase Inhibitors (Statins) [9]

Five compounds (*27, 146, 190, 173*, and *267*) are included. One (27) is a TN and one (*267*) was a TN in Fisher rats but FN in Sprague-Dawley rats as hepatocellular adenocarcinomas and thyroid follicular cell adenomas were observed in this strain. Compound (*146*) was a FP with hepatocellular hyperplasia at 3 months, while compound (*190*) is a TP with hepatocellular hyperplasia at 3 months (although only hypertrophy was observed in a 6-month study) and uterus endometrial polyps after 2 years. Compound (*173*) induced forestomach hyperplasia after 6 months, and thyroid follicular cell adenomas and carcinomas and forestomach squamous cell papillomas after 2 years. Whether or not all these tumors are related to the pharmacological action of statins is not completely certain, as discussed in Ref. (26). In fact, the outcome is that statins can be expected to be carcinogenic anyway. Therefore, we categorized the complete class as TP.

##### Remaining Metabolic Compounds

Six remaining compounds are included. An antidiabetic sulfonylureum derivative (*58*), a lipid replacement (*57*), a nicotinic acid derivative (*1*), a triglyceride-lowering compound (*43*), a PPAR-gamma agonist (*134*), and an aldose reductase inhibitor (*130*), are all TN. We gave the category TN to all these compounds as no other evidence for a proliferative effect on the basis of receptor stimulation could be found. A 3-betahydroxy derivative (*160*) and an SGLT-2 inhibitor (*151*) are FP, with adrenal gland hyperplasia in the cortex or kidney hyperplasia after 6 months, respectively. Thus far, no SGLT-2 inhibitor was associated with renal effects in rats (49, 50). An inhibitor of growth hormone (*258*) is FN with skin sarcomas and uterus endometrium carcinomas after 2 years.

#### Respiratory System

##### Anticholinergics [30]

Two anticholinergic compound (*61* and *128*) intended to be administered *via* the inhalation route are TN. Therefore, we categorized these compounds as TN based upon their pharmacology.

##### Adrenergic β_2_-Agonists [10]

Five compounds are included (*191, 199, 228, 280*, and *288*). The most common feature was ovarian leiomyomas as seen for four of the five compounds. For the RS, the association of mesovarian leiomyomas with β_2_-agonist is well described, although one compound (199) showed only thyroid adenoma. Even for recent long-acting β_2_-agonists such as indacaterol and vilanterol (51, 52), these tumors have been observed. A broader disturbance of the gender HM is clear from the mammary adenocarcinoma (*228*) and the effects on the pituitary (*191* and *288*). We categorized this class as TP based on pharmacology.

##### Corticosteroids [11]

Three compounds are included (*168, 170*, and *227*). Two compounds (*168* and *170*) are TP. Compound *168* showed mammary gland acinar hyperplasia at 6 months, and at 2 years, mammary gland fibromas, brain astrocytomas, and hepatocellular adenomas were observed. Compound *170* showed lymph node hyperplasia and pancreas islet cell hyperplasia at 6 months, and hepatocellular adenomas, pancreas acinar cell adenomas, mammary gland adenomas and adenocarcinomas, and bone osteomas and osteosarcomas were observed in the 2-year study. Compound *227* is a FN and showed pheochromocytomas, pancreatic acinar cell tumors, and skin fibrosarcomas. The relationship between the pharmacology of corticosteroids and the target organs observed is not easy to understand, but the wide distribution of corticosteroid receptors is in accordance with the broad list of organs bearing tumors, suggesting a broad pharmacological perspective. We categorized the corticosteroids as TP.

##### Histamine H_1_ Antagonists [48]

Seven compounds (*11, 12, 67, 154, 155, 195*, and *207*) are included, with three TN (*11, 12*, and *67*). Two FPs (*154* and *155*) showed mammary gland acinar cell hyperplasia at 3 months, while compound *155* showed islet cell hyperplasia and hepatocellular hypertrophy at 6 months. Only two compounds (*195* and *207*) were associated with tumors (FN). Compound *195* showed pheochromocytomas, and compound *207* showed thyroid follicular cell adenomas, hepatocellular adenomas, and carcinomas. It is likely that these effects are associated with the induction of liver enzymes, based upon the target organs and the presence of pituitary adenomas and carcinomas. In general, the class is categorized as TN.

##### Remaining Respiratory Compounds

Compound *184*, an antifibrotic agent, induced adrenal hyperplasia after 6 months, but liver adenoma and uterine adenocarcinoma after 2 years, making it histopathologically a TP. Pharmacologically, it could not be categorized, as no relationship is known to exist between the effects of the two endpoints. The effects are rather unspecific, and therefore, we finally decided to categorize it a TN. Compound 264 is a methylxanthine derivative is a FN with testis tumors and mammary fibroadenoma, rather than unspecific tumors, and therefore, we categorized this as TN. Compound 116 is a TN mast cell stabilizer.

#### Antimicrobial Agents

##### Antibiotics, Fluoroquinolones [13]

Three compounds (*231, 244*, and *263*) are all FN. The first showed renal adenomas and carcinomas, the second showed leukemia, and the third was associated with pancreatic neoplasms. As the primary pharmacology is not directed to a mammalian target, we categorized these compounds as non-mammalian target (NT).

##### Remaining Antibiotics

One compound (*158*), a bactericidal agent, is FP with forestomach hyperplasia at 6 and 12 months.

##### Antifungal Agents, Conazole Derivatives [14]

Three compounds (*182, 226*, and *236*) are included. The first (*182*) is a FN with skin, brain, testis, and mammary gland tumors. Compound *226* showed hepatocellular adenomas, and compound *236* showed a low incidence of soft tissue carcinomas. The classes of triazole antifungals do not have a mammalian target by definition, as discussed above. A specific effect on liver enzyme metabolism is described for related members of the class of antifungals (53). The first molecular event is not fully clear but might be the binding to a CYP450 subcategory. Conazoles induce hepatic cell proliferation in mice. We have categorized the antifungals as NT.

##### Remaining Antifungals

One compound (*2*) is a TN, while another (*279*) is a FN showing hepatocellular adenomas and carcinomas, as well as Leydig cell tumors. We categorized them all as NT.

##### Antimicrobials, Remaining Antimicrobials

Two AM are included, an antimalarial drug (*73*) and an antiparasital compound (*97*) (anti-lice). Both compounds are TN. As the primary pharmacology is not directed to a mammalian target, we categorized these compounds as NT.

##### Antivirals [32]

The antivirals (*18, 55, 60, 104, 123, 135, 171, 189, 218*, and *246*) do not belong to the same class and have different types of therapeutic use and modes of actions. Six compounds, an immunostimulant (*60*), a nucleoside-analog (*104*), a viral DNA polymerase inhibitor (*55*), a protease inhibitor (*18*), a nucleoside combination (*123*), and an anti-herpes compound (*135*), are all TN. Four compounds (*171, 189, 218*, and *246*) showed tumors. As antivirals do not have direct primary pharmacological targets in mammals, as discussed above, the variety of effects might be due to completely different mechanisms of action. For compound *246*, liver induction might have led to increased T3 metabolism, eventually resulting in thyroid tumors. A similar explanation is not possible for compounds *171* and *218*. In these individual cases, the potential human risk of the induction of tumors has been evaluated, and the relevance of these effects is negligible compared to the benefit of clinical treatment.

A CCR5 receptor antagonist (*246*) is FN with thyroid adenomas after 2 years. A guanosine analog (*171*) is FN with mammary gland adenocarcinomas after 2 years. A protease inhibitor (*189*) is TP and showed hepatocellular hyperplasia after 6 months and adrenal pheochromocytomas after 2 years. As none of the antiviral showed a clear connection to a mammalian pharmacological target, we just categorized them as NT.

#### Anti-inflammatory Compounds

##### Non-Steroidal Anti-inflammatory Drugs [19]

Twelve “classical” cyclooxygenase 1/2 inhibitors (*44, 45, 50, 64, 71, 74, 83, 91, 124, 129, 164*, and *260*) are included with 10 being TN. One (*164*) compound is a TP with kidney hyperplasia at 6 months, and after 2 years, adrenal pheochromocytomas and hyperplasia in the urinary bladder were observed. The histopathological changes and tumors induced cannot be directly explained. The adrenal pheochromocytomas are assumed to be not relevant for humans. For another compound (260), it is assumed that induction of CYP450 would lead to an increased testosterone metabolism, which leads *via* a feedback mechanism to Leydig cell adenomas. We have categorized this class as TN.

##### Cyclooxygenase-2-Inhibitors [31]

Four compounds (*108, 72, 206*, and *222*) are included. Two compounds (*72* and *108*) are TN. The two other compounds (*206* and *222*) are FN with hepatocellular adenomas and carcinomas. One compound (*222*) also showed thyroid follicular cell adenomas. The target organs suggest a relation with the liver-induced metabolism and not to a direct pharmacological response. This supports the negative properties of the NSAID (COX1/2-inhibitor) class, as discussed above. Based on this relationship, we categorized the class as TN.

##### Remaining Anti-inflammatory Compounds

Two compounds are included (*7* and *122*), which are both TN, and we categorized them the same.

#### Urinary Bladder

##### Anticholinergics [51]

Six compounds (*31, 51, 125, 268, 283*, and *287*) are included, with three (*31, 51*, and *125*) categorized as TN and three (*268, 283*, and *287*) as FN. Compound *268* was associated with benign uterine polyps and kidney papillomas, compound *283* with kidney sarcomas, and compound *287* with skin sarcomas, all with high dosages or incidental findings. The class was categorized as TN.

##### Remaining Urinary Compounds

A xanthine oxidase inhibitor (*153*) is FP with thyroid hyperplasia at 6 months. A β_3_-agonist (*79*) is TN. We have placed both in the category TN.

#### Bone Metabolism

##### Bisphosphonates [20]

Three compounds (*3, 33*, and *87*) are included and shown to be TN. Van der Laan et al. (26) described a pharmacological explanation for a positive finding, i.e., thyroid c-cell adenoma. This is not, however, confirmed in the present data, and we maintained the TN category.

##### Remaining Compounds Affecting Bone Metabolism

An isoflavone derivative (*234*) is a FN with pituitary gland adenomas and hepatocellular adenomas after 2 years. A calcium-mimetic (*28*) is TN. As the tumor targets for compound *234* are rather unspecific, we categorized both as TN.

#### Remaining Compounds

Four compounds (*39, 62, 109*, and *196*) remained pharmacologically unclassified. Three were TNs, a prostaglandin E2-analog (*39*), a CFTR potentiator (*62*), and a protein kinase C-beta inhibitor (*109*). A retinoid for topical administration (*196*) is a FN, with adrenal phaechromocytomas and thyroid follicular cell adenomas after 2 years. As the tumor effects are rather unspecific, we assigned a TN category to this compound and maintained the TN for the others.

## Discussion and Conclusion

The present retrospective study using data from rat subchronic (3- and/or 6-month) toxicity and 2-year carcinogenicity studies obtained from the non-clinical assessment reports, available at the Medicines Evaluation Board of the Netherlands, was performed to test independently a “weight-of-evidence approach,” expressed in the ICH Regulatory Notice Document (54) that was based on the “whole animal negative predictivity hypothesis” of Reddy et al. (25) and Sistare et al. (16). This might strengthen the assumption that the absence of any putative preneoplastic lesion in a subchronic study is associated with a negative outcome of a 2-year carcinogenicity study in rats, and therefore, no further long-term carcinogenicity study is needed. Furthermore, we have evaluated the role of the pharmacological properties in this respect.

In order to build evidence for the possibility of prediction, we used information that is commonly available at the end of Phase II in the development of human pharmaceuticals for designing a 2-year carcinogenicity study, including data on pharmacology and genotoxicity, and data from subchronic toxicity studies in rodents, as was undertaken earlier by Van der Laan et al. (26).

The dataset that we used has some overlap with the PFJ dataset as used by Van der Laan et al. (26), and we identified an overlap of 76 compounds. We decided to include these, as the application of more stringent criteria of putative neoplasm (see below) has led to differences in the categorization and to a higher number of FN.

### Role of Liver Hypertrophy versus Hyperplasia

A large number of pharmaceutical compounds induced greater (relative) liver weight, and the question arises about the role of this property in the weight-of-evidence approach. A greater liver weight may result from a wide variety of causes such as hyperplasia (of any of the resident cell types), hypertrophy, inflammation, fibrosis, abnormal storage of metabolism or cleavage products, neoplasia, and congestion (55–57). Typically, these changes do not occur in isolation, so in the absence of overt adverse changes such as inflammation, necrosis, or degeneration, it is important to recognize that an increase in liver weight may be induced by hypertrophy, hyperplasia, or a combination of the two (58). A xenobiotic that induces an increase in liver weight of 150% in a subchronic study might be considered to induce adverse effects in the context of dose setting for longer term studies but would not be considered to be adverse in the context of safety evaluation (59, 60).

In a survey of 139 chemicals used in the agrochemical industry, Carmichael et al. (59) demonstrated that a relative greater liver weight of ≥150% of control values was correlated with the induction of liver tumors in mice. In a similar review of rat studies, a less statistically significant relationship between liver weight and hepatocarcinogenesis was noted, whereby greater liver weight alone correctly predicted 8 of 11 liver carcinogens (but falsely predicted 26 as positives) and failed to predict 3 TPs (59).

Our findings demonstrate that treatment-related changes in organ weights, observed in a subchronic study with rats, are most likely non-specific and therefore should not be considered as a risk factor for neoplasia.

The histopathological diagnosis of hypertrophy can have various connotations, including a greater weight of the organ and an increase in the average size of the cells and even enzyme induction (functional hypertrophy). Allen et al. (61) evaluated the results for 111 chemicals tested by the National Toxicology Program. If they applied hepatocellular necrosis, hepatocellular hypertrophy, hepatocellular cytomegaly, and greater liver weight as predictors for carcinogenicity, greater liver weight appeared to be the most sensitive parameter. However, chemicals that produced liver tumors frequently induced multiple morphological changes. They concluded that the best single predictor of liver cancer in mice was hepatocellular hypertrophy. They found no FNs, but numerous FPs, in their evaluation.

In the present study, 33 compounds with a TN label showed an increase in hepatocellular hypertrophy in the subchronic study that was not accompanied by development of hepatocellular adenomas and/or carcinomas in the carcinogenicity study, whereas in only 7 cases was liver hypertrophy accompanied by the development of hepatocellular adenomas (3) or hepatocellular adenomas and carcinomas (4).

These observations support our starting point to classify hyperthrophy as an adaptive, rather than a putative, preneoplastic lesion. If we had assessed hepatocellular hypertrophy as an indicator for the development of hepatocellular tumors, 33 compounds would have been overpredicted as potential liver carcinogens (FP substances). This confirms that liver hypertrophy observed in a subchronic study is an unreliable predictor of carcinogenicity. This is in agreement with the conclusion from the 3rd International ESTP Expert Workshop (62) that hepatomegaly as a consequence of hepatocellular hypertrophy without histological or clinical pathological alterations indicative of liver toxicity is an adaptive reaction.

### Histopathological Evaluation

First, the predictivity was evaluated based upon the histopathological characterization.

*The negative predictivity*, the measure of the compounds evaluated that did not show any putative preneoplastic lesion in the subchronic studies and were negative in the carcinogenicity studies, was 60%, whereas the sensitivity, a measure of the subchronic study to predict positive carcinogenicity outcome, was only 24% (Table 2). In contrast, the specificity, the accuracy of the subchronic study to correctly identify non-carcinogens, was 88%. Based only on the absence of putative preneoplastic lesions in the subchronic study, 56% of the 2-year carcinogenicity studies could have been eliminated at the risk of 96 (33%) FN.

**Table 2:** **Predictivity of the subchronic toxicity study for the carcinogenicity of non-genotoxic pharmaceuticals**.**^a^**

		Carcinogenicity					
		Histopath. categoriz.		Pharmacol. categoriz.		Final categoriz.	
		Pos.	Neg.	Pos.	Neg.	Pos.	Neg.
Subchronic tox	Positive	31	19	62	21 (NT)	67	1
	Negative	96	143	14 (NC)	192	17	204

		**%**		**%**		**%**	

False negatives	=[96/(96 + 143 + 31 + 19)] × 100	33		5		6	
Negative predictivity^b^	=[143/(143 + 96)] × 100	60		93		92	
Positive predictivity^c^	=[31/(31 + 19)] × 100	62		75		98	
Sensitivity^d^	=[31/(31 + 96)] × 100	24		82		80	
Specificity^e^	=[143/(143 + 19)] × 100	88		90		99	

*^a^The subchronic (3-month) study results were used to categorize a compound as true positive (TP), false positive (FP), false negative (FN), and true negative (TN)*.

*^b^Ability to predict non-carcinogens: [TN/(TN + FN)] × 100*.

*^c^Ability to predict rat carcinogens: [TP/(TP + FP)] × 100*.

*^d^Ability to detect rat carcinogens: [TP/(TP + FN)] × 100*.

*^e^Ability to detect non-carcinogens: [TN/(TN + FP)] × 100*.

*The positive predictivity* (62%) is the percentage of compounds that showed putative preneoplastic changes in the subchronic study and caused treatment-related tumors in the 24-month carcinogenicity study.

Thirty-one substances were classified as TP. However, for only 13 compounds, the putative preneoplastic lesions developed in the same organs as the tumors. Eighteen compounds induced hyperplastic lesions in another organ than the organ where the tumors occurred. This observation is in agreement with the conclusion of Reddy et al. (25) that the whole animal approach assumes that preneoplastic changes at any organ will be indicative for an increase in tumor incidence in that organ or in any organ at a distant site. A closer look at the organs (Table S1 in Supplementary Material: compounds 163–193) illustrates the importance of physiological relationships between organs. Several compounds induce their own metabolism in the liver and, as a consequence, enhance the enzymes responsible also for the metabolism of hormones, such as T3 and testosterone/estradiol, lowering their concentration. This decrease in hormone concentration leads to a feedback responses to the pituitary, resulting in enhanced secretion of thyroid-releasing hormone and/gonadotropin-releasing hormone, which in turn may lead to the development of tumors.

### False Positive Compounds

Nineteen chemicals induced putative preneoplastic (hyperplastic) histopathological changes in the liver, thyroid, kidneys, mammary glands, pancreas, stomach, adrenals, and incidentally in other organs, whereas no tumors occurred in the carcinogenicity study, neither in the same organ nor in an organ at a distant site. This observation supports the conclusion of Jacobs (24), based on an evaluation of 60 pharmaceuticals, that various short-term indicators for carcinogenicity, such as hyperplasia, do not always result in tumors in that tissue, although such putative preneoplastic histopathological lesions are generally considered a sign of potential concern for carcinogenicity.

Woutersen et al. (63) performed a retrospective study and evaluated the subchronic (3 months) studies of 163 non-genotoxic chemicals with the aim to predict the tumor outcome of 24-month rat carcinogenicity studies. In this study, the negative predictivity, a measure to predict a negative carcinogenicity outcome, amounted to 97%, whereas the sensitivity, a measure to predict a positive carcinogenicity outcome, was only 5%. Overall, this study supports the concept that chemicals showing no histopathological risk factors for neoplasia in a subchronic study in rats may be considered non-carcinogenic and do not require further testing in a carcinogenicity study. The findings observed in the present paper with TP and FP compounds are in agreement with the conclusion of Reddy et al. (25) that more research is needed in order to achieve understanding of the biological links between putative preneoplastic lesions observed in a subchronic study and tumors developing at distant organ sites in the carcinogenicity study. The concept of adverse outcome pathways is helpful in this respect, defining as the first step the molecular event and then subsequent steps leading to the final outcome of tumors in different organs.

### False Negative Compounds

Since it is generally accepted that the intention of screening assays should be conservative, it is most important that the number of FNs with respect to human carcinogens should be as low as possible. In the present study, 96 compounds were classified as FN because they did not show putative preneoplastic lesions in the 3- and/or 6-month study but caused treatment-related tumors in the carcinogenicity study. These compounds are of concern with regard to the acceptability of the negative predictivity of the whole animal approach stating that the absence of evidence of putative preneoplastic lesions in all tissues in the 3- and/or 6-month study may serve as a strong negative predictor of tumor outcome in the carcinogenicity study.

When we evaluate these FN substances histopathologically, 77 of the 96 FN substances appeared to induce benign tumors or benign and malignant tumors, which are considered not relevant for the human situation (36): acinar pancreatic tumors and islet cell neoplasia (36); pheochromocytomas of the adrenal medulla (64); forestomach tumors (65, 66); hepatocellular tumors induced by peroxisome proliferators (48, 67–69); fibroadenomas of the mammary gland (70); pituitary tumors (adenohypophysis tumors) (71); Leydig cell (interstitial cell) tumors of the testes (72, 73); thyroid follicular cell tumors (74–76); and urinary bladder tumors (75, 77–80) and uterus tumors (endometrial stromal polyps) (81). That means that 19 FN substances still remain using this approach.

### Impact of Pharmacology

To evaluate further the remaining 19 FN substances, we compared this number with the outcome of an evaluation integrating the pharmacological properties of the compounds. We have tested the hypothesis that a pharmacological explanation, as known for several years (30), and integrated in the histopathological approach recently (26), will help to reduce the number of FNs.

To study the role of the pharmacology in relation to histopathology, we have evaluated whether the mode of action is related to a proliferative mechanism or only to non-proliferative mechanisms. We have selected those classes that are clearly related to induction of tumors as positive classes (Table 3) and classes not related to induction of tumors as negative classes (Table 4). A series of classes with mixed outcome have been evaluated in relation to literature, and for each pharmacological class, a clear outcome of “positive” or “negative” has been chosen (Table 5), meaning a high or low percentage of rat carcinogens, respectively. For the remaining individual compounds, we have taken into consideration the specificity of the tumors, i.e., those tumors likely to be associated with a change in metabolism of hormones (thyroid, or testosterone, or calciferol) are not related to a specific pharmacological mechanism. The compounds are, therefore, categorized as TN.

**Table 3:** **Classes with high percentage of rat carcinogens (positive classes)**.

	Class	Total number of compounds	Compounds with tumors	Proposed final category
**Related to direct pharmacology**				
1	CNS, DA_2_ agonists	4	4 (100%)	TP
2	CNS, DA_2_ antagonists	4	3 (75%)	TP
3	HM, GnRH agonists	4	4 (100%)	TP
4	HM, estrogen agonists	2	2 (100%)	TP
5	HM, selec. estrogen receptor mod.	2	2 (100%)	TP
6	HM, dual 5-reductase inhibitors	2	2 (100%)	TP
7	HM, progestogen (combinations)	3	2 (67%)	TP
8	MB, HMG-CoA reductase inhibitors	5	3 (60%)	TP
9	MB, fibrates	3	2 (67%)	TP
10	RS, adrenergic β_2_ agonists	5	5 (100%)	TP
11	RS, corticosteroids	3	3 (100%)	TP
12	GI, PP inhibitors	4	3 (75%)	TP
**Not related to direct pharmacology**				
13	Antibacterial, fluoroquinolones	3	3 (100%)	NT
14	Antifungal triazole derivatives	3	3 (100%)	NT
15	CVS, calcium antagonists	12	8 (67%)	TN
16	CVS, loop diuretics	4	3 (75%)	NC
17	CNS, 5HT_2_ antagonists	2	2 (100%)	NC
18	GI, 5HT_4_ agonist	3	2 (67%)	TN

**Table 4:** **Classes with low percentage of rat carcinogens (negative class)**.

	Class	Total number of compounds	Compounds with tumors	Proposed final category
19	AI, NSAIDs	12	2 (17%)	TN
20	BM, bisphosphonates	3	–	TN
21	CVS, angiotensin II antag.	5	–	TN
22	CVS, class 1C antiarrhytmics	2	–	TN
23	CVS, endothelin antagonists	2	–	TN
24	CVS, vasopressin-2 agonists	2	–	TN
25	CVS, adrenergic β-antagonists	13	3 (21%)	TN
26	CNS, μ-opioid antagonists	2	–	TN
27	CNS, SSRIs	7	1 (14%)	TN
28	MB, DPP4 inhibitors	4	–	TN
29	IS, immunosuppressives	6	–	TN
30	RS, anticholinergics	2	–	TN

**Table 5:** **Classes with medium percentage of rat carcinogens (mixed outcome class)**.

	Class	Total number of compounds	Compounds with tumors	Proposed final category
31	AI, COX-2 inhibitors	4	2 (50%)	TN
32	Antivirals	10	4 (40%)	NT
33	CVS, ACE inhibitors	9	5 (55%)	TN
34	CVS, adrenergic α_1_ antag.	8	3 (38%)	TN
35	CVS, adrenergic α_1_ agonist	2	1 (50%)	TN
36	CVS, adrenergic α_2_ agonist	3	1 (33%)	TN
37	CVS, anticoagulant	2	1 (50%)	TN
38	CVS, imidazoline agonists	2	1 (50%)	TN
39	CVS, Na-channel blockers	3	1 (33%)	TN
40	CVS, platelet aggreg. inhib.	2	1 (50%)	NC
41	CVS, PDE_3_ inhibitors	2	1 (50%)	TN
42	CNS, μ-opioid agonists	3	1 (33%)	TN
43	CNS, 5HT_1b/d_ agonists	4	2 (50%)	TN
44	CNS, 5HT_3_ antagonists	2	1 (50%)	TN
45	CNS, benzodiazepines	5	2 (40%)	TN
46	CNS, antiepileptic, Na-channel blocker	6	2 (33%)	TN
47	CNS, SNRIs	4	2 (50%)	TN
48	RS, histamine H_1_ antag.	7	2 (28%)	TN
49	GI, histamine H_2_ antag.	4	2 (50%)	TN
50	MB, α-glycosidase inhibitors	2	1 (50%)	TN
51	UB, anticholinergics	5	3 (60%)	TN
52	IS, immunomodulators	2	1 (50%)	NC

Based upon pharmacology, combined with a previous histopathological categorization, we have given a final category in a separate column. This final category is applicable only to those classes for which at least two compounds are present in the dataset.

We have separated the compounds without a direct pharmacological effect in mammalian tissue from the other compounds.

Table 3 contains a list of pharmacological classes with a high percentage of rat carcinogens (positive classes). In line with the earlier overview (26), we can differentiate between carcinogenicity directly related to pharmacology and carcinogenicity not related to pharmacology. Antibiotics, antifungals, and antivirals are developed to act against specific mechanisms in their target organism. Of course, it is possible that off-target effects exist in mammals. Apparently, this is true for the metabolic enzymes in liver. We have chosen, therefore, to give a separate classification to all compounds without a non-mammalian target (NT).

Based upon the identity of the organs bearing tumors (i.e., associated with liver, thyroid, adrenal, and testis), we have put the antibacterials, antifungals, and antivirals in the same category.

The calcium antagonists, the loop diuretics, and the GI 5HT_4_ agonists are in the list of “not related to pharmacology.”

### Combined Evaluation of Histopathology and Pharmacology

In Table 1, we have incorporated the pharmacology-based categorization in the column labeled as Cat. Ph, by taking the pharmacology as an additional factor (Table 1, fifth column). In this way, we categorized most of the compounds as TP (if belonging to a positive class) or TN, as explained above.

A combination of the histopathological categorization of the compounds together with the pharmacological categorization of the pharmaceuticals gave the following overall results:

- Twenty-one pharmaceuticals that do not have a mammalian target (8FN, 1FP, 9TN, and 3TP) are included.
- Fifty-two of the 96 FN compounds were recategorized as TNs based on pharmacology; 27 were recategorized as TPs; in addition to the 8 without mammalian target, 9 were not recategorized due to an unknown relationship between pharmacology and carcinogenicity.
- Fourteen FP compounds were recategorized to TN based on pharmacology; five were recategorized as TP. One remained FP, as there is no pharmacological target.
- One hundred twenty-four TNs remained TN, while 14 were categorized as TP. Five were not categorized (NC).
- From the 41 TP compounds, 11 were recategorized as TN, while 17 remained TP on the basis of their pharmacology. Two could not be categorized, and three had no mammalian target.
- Finally, 14 out of 289 (5%) pharmaceuticals evaluated in the present retrospective study have not been recategorized. Nine are FN, and five are TN.
- After recategorization, based on both histopathology and pharmacology, the number of FN compounds was reduced to only 17 out of 289 (6%). The negative predictivity amounted to 92%; the positive predictivity to 98%, and the sensitivity was 80%, whereas the specificity amounted to 99% (TP: 67; FP: 1; FN: 17; and TN: 204) (see Table 6).

**Table 6:** **Summarizing table based upon final categorization**.

	Histopathological categories (Table S3 in Supplementary Material)	Pharmacological categories (Table S4 in Supplementary Material)	Final categories (Table 1)	
TN	143	192 (47^a^)	204	No preneoplastic signals in subchronic studies, no pharmacological signals, no carcinogenicity
TP	31	62 (5^a^)	67	Preneoplastic signals in subchronic studies, pharmacological signals, carcinogenicity
FN	96		17	Not conclusive
FP	19		1	
NT		21		
NC		14		
Total	289	289	289	

*^a^Single-in-class decisions*.

*NC, non-categorizable based on pharmacological target; NT, non-mammalian target in mammalian tissue; FN, false negative; FP, false positive; TN, true negative; TP, true positive*.

When the FN compounds that gave rise to tumors generally considered not relevant for human risk assessment, and those categorized as TN based on the pharmacological analyses, were moved from the FN category to the TN category, the negative predictivity of the 3- and/or 6-month study for the absence of carcinogenicity (the ability to predict non-carcinogens) amounts to 96% and the specificity (the ability to detect non-carcinogens) to 99% (Table 3).

The analysis of this paper clearly shows that adding pharmacological properties as an additional factor of potential carcinogenicity gives a good prediction, reducing the number of FN substantially, which has consequences for the risk assessment of these pharmaceuticals.

## Conclusion

Our results demonstrated that subchronic (3- and/or 6-month) studies, in combination with knowledge of pharmacological properties, could appropriately categorize non-genotoxic pharmaceutical into two categories:

(i) unlikely to be carcinogenic in rats if (1) no histopathological risk factor for neoplastic lesions observed in the subchronic study in any tissue, and (2) general absence of systematic, specific carcinogenicity in the pharmacological class.
(ii) likely carcinogenic in rats if (1) putative preneoplastic lesions observed in the subchronic study may give rise to a type of tumor in the rat 24-month carcinogenicity study that is irrelevant for humans; therefore, a carcinogenicity study has no additional value; (2) this is confirmed by the results from the pharmacological class.

We should keep in mind that the real focus is on the prediction of carcinogenicity in humans, and we cannot quantify the full translational value of the rodent carcinogenicity study. However, overall, the results of this retrospective study support the whole animal approach as proposed by Reddy et al. (25) and Sistare et al. (16), especially with respect to the negative outcome of the subchronic studies as prediction for a negative outcome of a carcinogenicity study. Moreover, the results (predictivity) are consistent with the recent and similar investigation on chemicals (63). Furthermore, the data show the added value of the pharmacological evaluation of compounds in relation to potential class effects, both in the negative and positive direction. This evaluation strongly enhances the prediction of a possible impact for rodents and eventually for an extrapolation of the carcinogenic risk to humans. The outcome can be used to further prevent conducting unnecessary carcinogenicity studies. For most of the pharmacological mechanisms, it is well known that the non-genotoxic mode of action carries no risk of carcinogenicity for the human situation because of important species-related differences between rodents and humans.

In this way, the pharmacological analysis confirms the approach recently published by Van der Laan et al. (26) and reflected in the ICH Regulatory Notice Document to incorporate the pharmacological properties in predicting a positive and a negative outcome of a 2-year carcinogenicity study.

A high negative and a high positive predictivity of the carcinogenic potential of a pharmaceutical compound based on the findings in subchronic toxicity studies in rats, combined with knowledge of the pharmacological class, should result in waiving the need for conducting 2-year rat carcinogenicity studies, which will lead to a reduction in the numbers of animals used for scientific purposes and will save time and expense for drug development.

The dataset used for this analysis gives important opportunities for further research. As in addition to rat data, also mouse data are included, we can have a better understanding of the different outcomes between rats and mice, as discussed previously by Van Oosterhout et al. (27) and Friedrich and Olejniczak (15). Their reports point to the low regulatory relevance of lifetime mouse studies, and potential reduction of 2-year rat studies as proposed in the ICH RND (54), should at least be accompanied by measures with respect to 18- or 24-month mouse studies. Further discussion is recommended on the predictive value of the involvement of cytochrome *p* 450ies in the induction of cell proliferation in liver or other organs such as thyroid gland and testis. These and other questions require further analysis of the studies in this database.

## Author Contributions

WB, LW, and AS conducted the data retrieval and filled the database. ES translated the database in an accessible spreadsheet. CK contributed to the design of the study and criticized the manuscript. RW and JL have equally contributed to the writing and the underlying concepts, with RW responsible for histopathological aspects and JL for the pharmacological and regulatory aspects.

## Conflict of Interest Statement

The authors declare that the research was conducted in the absence of any commercial or financial relationships that could be construed as a potential conflict of interest.

## Abbreviations

AB, antibiotics; ac, adenocarcinoma; ad, adenoma; AF, antifungal agents; adr, adrenals; astr, astrocytoma; AV, antivirals; bheam, benign hemangioma; bpha, benign pheochromocytoma; bm, bone marrow; bo, bone; br, brain; bthym, benign thymoma; ca, carcinoma; ce, cecum; col, colon; fad, fibroadenoma; FN, false negative; FP, false positive; gca, granulosa cell adenoma; hrt, heart; hsyst, hematopoietic system; islet, islet of Langerhans; kid, kidneys; leio, leiomyoma; leu, leukemia; li, liver; lip, lipoma; ln, lymph nodes; lu, lungs; lymph, lymphoma; mam, mammary glands; mel, melanoma; mes, mesentrium; most, malignant osteoma = osteosarcoma; mpha, malignant pheochromocytoma; NC, non-categorizable based on pharmacological target; NOS, not otherwise specified; NT, non-mammalian target in mammalian tissue; ova, ovaries; pan, pancreas; pap, papilloma; parathy, parathyroids; pit, pituitary; pros, prostate; sar, sarcoma; SCC, squamous cell carcinoma; schwan, schwannoma; SCP, squamous cell papilloma; soft t, soft tissue; spl, spleen; stom, stomach; tes, testes; thyr, thyroid; thym, thymus; TN, true negative; TP, true positive; tu, tumor; UGT, urogenital tract; ut, uterus; zymgl, Zymbal’s gland; ZZ, remaining compounds.
