# Supplementary material for: Prediction of the Carcinogenic Potential of Human Pharmaceuticals Using Repeated Dose Toxicity Data and Their Pharmacological Properties
Source: Front Med (Lausanne). 2016 Oct 14;3:45. doi: 10.3389/fmed.2016.00045 (PMC5063850; doi:10.3389/fmed.2016.00045)
Supplement: Supplementary file 2 [file table_2.pdf]

Table S2 (Supplementary Material) Summary of the observations in the sub-chronic and carcinogenicity studies, sorted by mode of action								
#	Mode of Action	Cat. His	Cat. Ph.	Fin. cat.	Weight	Sub-chronic		Carcinogenicity
						HT	HP	
231	AB, Fluoroquinolone	FN	NT	FN	col ; kid	-	-	hsyst leu
244	AB, Fluoroquinolone	FN	NT	FN	-	-	-	pan tu
263	AB, Fluoroquinolone	FN	NT	FN	ce ; hrt ; li ; spl ; adr ; ova	-	-	kid ac
158	AB, remaining, bactericidal	FP	NT	FP	li ; spl ; kid ; thyr	-	stom hp; ut hp; stom hp	-
182	AF, conazole derivative	TP	NT	TP	li ; kid ; spl ; br ; ova ; thyr	-	thyr hp	tes tu; br astr; skin mel; mam ac
226	AF, conazole derivative	FN	NT	FN	li	li ht	-	li ad
236	AF, conazole derivative	FN	NT	FN	adr ; li ; hrt ; kid ; thy ; lu ; spl ; pan ; br ; gon ; ova	adr ht	-	soft t sar
279	AF, remaining, allylamine derivative	FN	NT	FN	hrt ; adr	-	-	tes tu; li ad; li ac
2	AF, remaining, benzimidazole	TN	NT	TN	-	-	-	-
108	AI, COX2 inhibitor	TN	TN	TN	-	-	-	-
72	AI, COX2-inhibitor	TN	TN	TN	-	-	-	-
206	AI, COX2-inhibitor	FN	TN	TN	li	-	-	li ac
222	AI, COX2-inhibitor	FN	TN	TN	-	li ht; thyr ht	-	thyr ad; li ad
44	AI, NSAID	TN	TN	TN	kid	-	-	-
45	AI, NSAID	TN	TN	TN	-	-	-	-
50	AI, NSAID	TN	TN	TN	kid ; spl	-	-	-
64	AI, NSAID	TN	TN	TN	-	-	-	-

74	AI, NSAID	TN	TN	TN	-	-	-	-
83	AI, NSAID	TN	TN	TN	li ; kid	-	-	-
91	AI, NSAID	TN	TN	TN	hrt ; adr ; kid	-	-	-
124	AI, NSAID	TN	TN	TN	-	-	-	-
129	AI, NSAID	TN	TN	TN	-	-	-	-
164	AI, NSAID	TP	TN	TN	-	-	kid hp; UGT hp	adr bpha
260	AI, NSAID	FN	TN	TN	-	-	-	tes ad;
71	AI, NSAID,	TN	TN	TN	-	-	-	-
7	AI, remaining,	TN	TN*	TN	li;	-	-	-
122	AI, remaining, cytokine-modulat	TN	TN*	TN	-	-	-	-
73	AM, remaining, antimalarial	TN	NT	TN	-	-	-	-
97	AM, remaining, Antiparasite.	TN	NT	TN	-	-	-	-
123	AV,	TN	NT	TN	-	-	-	-
246	AV, CCR5 receptor antagonist	FN	NT	FN	-	thyr ht	-	thyr ad
171	AV, Guanosine analogue	TP	NT	TP	-	pit ht	tes hp	mam ac; skin sar
218	AV, hepatitis B-inhibitor	FN	NT	FN	-	-	-	pan ad; pan ac; li ad; li ac; Zymgl ca; br gli
135	AV, herpes genitalis	TN	NT	TN	-	-	-	-
60	AV, immunostimulant	TN	NT	TN	li ; kid ; adr	-	-	-
104	AV, Nucleoside inhibitor	TN	NT	TN	-	-	-	-
18	AV, protease inhibitor	TN	NT	TN	-	-	-	-
189	AV, protease inhibitor	TP	NT	TP	-	thyr ht	li hp; kid hp	adr bpha
55	AV, viral DNA polymerase inhibitor	TN	NT	TN	-	-	-	-
3	BM, bisphosphonate	TN	TN	TN	-	-	-	-
33	BM, bisphosphonate	TN	TN	TN	-	-	-	-
87	BM, Bisphosphonate,	TN	TN	TN	thyr ; parath	bo ht	-	-
28	BM, remaining, calcium-mimetic	TN	TN*	TN	-	-	-	-

234	BM, remaining, Isoflavone	FN	TN*	TN	-	-	-	pit ad; li ad
4	CNS, 5-HT1b/d agonist	TN	TN	TN	-	thyr ht; li ht	-	-
277	CNS, 5-HT1b/d agonist	FN	TN	TN	-	-	-	adr bpha; tes ad
107	CNS, 5-HT1b/d agonist,	TN	TN	TN	-	-	-	-
181	CNS, 5-HT1b/d agonist,	TP	TN	TN	kid	-	epi hp; tes hp	thyr ad; pit ad; thy bthym
220	CNS, 5HT2 antagonist	FN	NC	FN	-	-	-	li ad
185	CNS, 5-HT2 antagonist	TP	NC	TP	li	thyr ht; mam ht	mam hp	thyr ad; mam ac
95	CNS, 5-HT3 antagonist	TN	TN	TN	-	-	-	-
176	CNS, 5-HT3 antagonist	TP	TN	TN	-	-	-	li ad; li ac
24	CNS, antiepileptic, Na-channel blocker	TN	TN	TN	-	li ht	-	-
49	CNS, antiepileptic, Na-channel blocker	TN	TN	TN	-	-	-	-
65	CNS, antiepileptic, Na-channel blocker	TN	TN	TN	-	li ht	-	-
66	CNS, antiepileptic, Na-channel blocker	TN	TN	TN	-	li ht	-	-
183	CNS, antiepileptic, Na-channel blocker	TP	TN	TN	kid ; adr	li ht	kid hp	li ac
239	CNS, antiepileptic, Na-channel blocker	FN	TN	TN	adr ; pit ; kid ; li	-	-	adr bpha
5	CNS, Benzodiazepine	TN	TN	TN	-	-	-	-
204	CNS, Benzodiazepine	FN	TN	TN	-	-	-	thyr ad; thy lymph; ut schwan
248	CNS, benzodiazepine	FN	TN	TN	-	li ht	-	thyr ad
142	CNS, benzodiazepine-like hypnotic	TN	TN	TN	-	-	-	-
143	CNS, benzodiazepine-like hypnotic	TN	TN	TN	spl ; li ; kid ; tes ; hrt ; pit	li ht	-	-
274	CNS, DA2 agonist	FN	TP	TP	adr	li ht	-	tes ad; skin fibr
245	CNS, DA2 agonist	FN	TP	TP	-	-	-	tes ad; tes ca
265	CNS, DA2 agonist	FN	TP	TP	-	-	-	pit ad; ut ac
270	CNS, DA2 agonist	FN	TP	TP	-	-	-	tes ad

273	CNS, DA2-antagonist	FN	TP	TP	-	-	-	islet ad; mam ac; pit ad
163	CNS, DA2-antagonist, Benzamide,	TP	TP	TP	-	-	mam hp	pan ad; pan ac; adr bpha; mam ca; pit ca
188	CNS, DA2-antagonist, DA3 antagonist	TP	TP	TP	li	-	lu hp	mam ca
59	CNS, DA2-antagonist/5HT antagonist	TN	TP	TP	-	-	-	-
84	CNS, Opioid, mu-agonist	TN	TN	TN	-	-	-	-
205	CNS, Opioid, mu-agonist	FN	TN	TN	-	-	-	tes tu; hsyst leu
132	CNS, Opioid, mu-agonist, anticholinergic	TN	TN	TN	-	-	-	-
85	CNS, Opioid, mu-antagonist	TN	TN	TN	-	-	-	-
86	CNS, Opioid, mu-antagonist	TN	TN	TN	-	-	-	-
75	CNS, Opioid, remaining, kappa agonist	TN	TN*	TN	-	-	-	-
22	CNS, remaining 5HT, 5-HT1-agonist	TN	TN*	TN	-	-	-	-
56	CNS, remaining, acetylcholinesterase inhib	TN	TN*	TN	-	sgl ht	-	-
230	CNS, remaining, alpha2-delta agonist	FN	NC	FN	-	-	-	pan ac; pan ad; tes ad; ut polyp
96	CNS, remaining, AMPA Glutamate antagonist	TN	TN*	TN	-	-	-	-
251	CNS, remaining, antidepressant	FN	NC	FN	-	li ht; thyr ht	-	thyr ac; mam ca; li ad; li ac
106	CNS, remaining, cannabinoid antagonist	TN	TN*	TN	-	-	-	-
213	CNS, remaining, Carbonic anhydrase inhibitor	FN	TP*	TP	-	-	-	UGT pap
217	CNS, remaining, COMT-inhibitor	FN	NC	FN	adr	-	-	kid ad; kid ac
20	CNS, remaining, DA-NA uptake inhibitor	TN	TN*	TN	li ; adr ; thyr	li ht	-	-
177	CNS, remaining, Electron transporter	TP	TP*	TP	-	-	stom hp	Squamous cell and basal carcinomas
118	CNS, remaining, GABA-enhancer	TN	TN*	TN	-	-	-	-
138	CNS, remaining, GABA-metab. inhib	TN	TN*	TN	-	-	-	-

81	CNS, remaining, MAO-A inhibitor	TN	TN*	TN	lu ; kid ; thyr ; tes ; ova	-	-	-
102	CNS, remaining, MAO-B inhibitor	TN	TN*	TN	-	li ht	-	-
197	CNS, remaining, melatonin receptor agonist	FN	TN*	TN	-	-	-	li ad; li ac
136	CNS, Remaining, Nicotine agonist	TN	TN*	TN	-	-	-	-
223	CNS, remaining, NMDA-antagonist	FN	TN*	TN	-	-	-	tes ad
261	CNS, remaining, nootropic drug	FN	TN*	TN	-	-	-	adr bpha
103	CNS, SNRI	TN	TN	TN	-	-	-	-
137	CNS, SNRI	TN	TN	TN	-	-	-	-
250	CNS, SNRI	FN	TN	TN	-	li ht	-	thyr ad
276	CNS, SNRI	FN	TN	TN	kid	-	-	tes ad
29	CNS, SSRI	TN	TN	TN	-	-	-	-
35	CNS, SSRI	TN	TN	TN	-	-	-	-
54	CNS, SSRI	TN	TN	TN	-	-	-	-
112	CNS, SSRI	TN	TN	TN	kid	li ht	-	-
157	CNS, SSRI	FP	TN	TN	-	li ht	li hp	-
262	CNS, SSRI	FN	TN	TN	li	-	-	ln lymph
88	CNS, SSRI, 5-HT antagonist	TN	TN	TN	-	-	-	-
15	CVS, ACE inhibitor	TN	TN	TN	-	-	-	-
37	CVS, ACE inhibitor	TN	TN	TN	-	-	-	-
69	CVS, ACE inhibitor	TN	TN	TN	-	-	-	-
117	CVS, ACE inhibitor	TN	TN	TN	kid	kid ht	-	-
174	CVS, ACE inhibitor	TP	TN	TN	thyr	-	kid hp	pit ad; br ac; mes lip; pit ac
186	CVS, ACE inhibitor	TP	TN	TN	-	kid ht	kid hp	ln bhaem
208	CVS, ACE inhibitor	FN	TN	TN	kid ; li	kid ht	-	tes tu
266	CVS, ACE inhibitor	FN	TN	TN	-	-	-	thyr ac
271	CVS, ACE inhibitor	FN	TN	TN	-	-	-	kid ad

285	CVS, ACE inhibitor	FN	TN	TN	-	-	-	mam fad
233	CVS, ACE-inhibitor	FN	TN	TN	kid	-	-	thyr ad; ut polyp
159	CVS, Alpha1 agonist	FP	TN	TN	-	-	mam hp	-
249	CVS, Alpha1 agonist	FN	TN	TN	-	-	-	tes ad
19	CVS, Alpha1 antagonist	TN	TP	TP	kid ; br ; tes	-	-	-
34	CVS, Alpha1 antagonist	TN	TP	TP	-	-	-	-
133	CVS, Alpha1 antagonist	TN	TP	TP	-	-	-	-
144	CVS, Alpha1 antagonist	FP	TP	TP	-	-	mam hp	-
192	CVS, Alpha1 antagonist	TP	TP	TP	-	li ht; vag ht	li hp; mam hp	thyr ad; thyr ac
193	CVS, Alpha1 antagonist	TP	TP	TP	-	-	mam hp	mam ad; hsyst leu
278	CVS, Alpha1 antagonist	FN	TP	TP	br ;li ; kid ; hrt	-	-	adr bpha; mam ac
161	CVS, Alpha1 antagonist and 5-HT1A	FP	TN	TP	-	-	bm hp	-
145	CVS, Alpha2 agonist	FP	TN	TN	-	-	thy hp	-
149	CVS, Alpha2 agonist	FP	TN	TN	-	-	islet hp	-
167	CVS, Alpha2 agonist, indicatie ocular	TP	TN	TN	-	int ht	int hp	pan ac; thyr ad; mam ad
13	CVS, Angiotensin II antagonist	TN	TN	TN	-	-	-	-
23	CVS, Angiotensin II antagonist	TN	TN	TN	-	kid ht	-	-
40	CVS, Angiotensin II antagonist	TN	TN	TN	-	-	-	-
156	CVS, Angiotensin II antagonist	FP	TN	TN	-	-	kid hp	-
162	CVS, Angiotensin II antagonist	FP	TN	TN	-	kid ht	kid hp	
10	CVS, anticoagulant	TN	TN	TN	-	-	-	-
289	CVS, anticoagulant	FN	TN	TN	-	-	-	pan ad/ca
14	CVS, Beta antagonist	TN	TN	TN	-	-	-	-
16	CVS, Beta antagonist	TN	TN	TN	-	-	-	-
17	CVS, Beta antagonist	TN	TN	TN	hrt ; li	-	-	-

25	CVS, Beta antagonist	TN	TN	TN	pit ; lu ; hrt ; spl ; kid ; adr ; tes ; ova ; br	-	-	-
26	CVS, Beta antagonist	TN	TN	TN	-	-	-	-
126	CVS, Beta antagonist	TN	TN	TN	-	-	-	-
127	CVS, Beta antagonist	TN	TN	TN	-	-	-	-
147	CVS, Beta antagonist	FP	TN	TN	-	adr ht	thyr hp	-
203	CVS, Beta antagonist	FN	TN	TN	tes ; adr ; li	-	-	pit tu
219	CVS, Beta antagonist	FN	TN	TN	kid	-	-	skin SCP
243	CVS, Beta antagonist	FN	TN	TN	thyr ; li ; adr ; kid	-	-	li ad
148	CVS, Beta antagonist /alpha-1 blocker	FP	TN	TN	li	-	li hp	-
255	CVS, Beta antagonist,	FN	TN	TN	-	-	-	spl bhaem
9	CVS, Calcium antagonist	TN	TN	TN	hrt ; kid	adr ht	-	-
90	CVS, Calcium antagonist	TN	TN	TN	spl ; kid ; ova ; hrt ; li ; adr ; br	-	-	-
92	CVS, Calcium antagonist	TN	TN	TN	-	-	-	-
93	CVS, Calcium antagonist	TN	TN	TN	-	-	-	-
165	CVS, Calcium antagonist	TP	TN	TN	li	li ht	ln hp; thyr hp	thyr ad
172	CVS, Calcium antagonist	TP	TN	TN	-	-	col hp	mam fad; adr bpha; tes ad; pit ad; mam ac; pit ca
200	CVS, Calcium antagonist	FN	TN	TN	-	-	-	ut polyp
235	CVS, Calcium antagonist	FN	TN	TN	-	-	-	tes ad
237	CVS, Calcium antagonist	FN	TN	TN	ova	-	-	tes ad
240	CVS, Calcium antagonist	FN	TN	TN	-	adr ht	-	mam fad; pit ad
256	CVS, Calcium antagonist	FN	TN	TN	-	-	-	thyr ad; thyr ac
247	CVS, Calcium antagonist.	FN	TN	TN	li ; hrt	-	-	ut polyp; oral SCC

38	CVS, class 1C antiarrhythmic	TN	TN	TN	thyr ; li	-	-	-
53	CVS, class 1C antiarrhytmic	TN	TN	TN	hrt ; li	-	-	-
6	CVS, endothelin antagonist	TN	TN	TN	-	li ht; int ht; adr ht; mam ht	nose hp; bm hp	-
115	CVS, endothelin antagonist	TN	TN	TN	-	-	-	-
105	CVS, Imidazoline agonist	TN	TN	TN	adr ; tes	-	-	-
252	CVS, Imidazoline agonist	FN	TN	TN	-	-	-	adr tu
98	CVS, Loop diuretic	TN	NC	TN	-	-	-	-
229	CVS, Loop diuretic	FN	NC	FN	-	-	-	thyr ad; pit ad
253	CVS, Loop diuretic	FN	NC	FN	-	-	-	tes ad; ut ac
284	CVS, Loop diuretic	FN	NC	FN	-	-	-	kid ac; kid ad
100	CVS, Na-channel block	TN	TN	TN	-	-	-	-
101	CVS, Na-channel block	TN	TN	TN	-	li ht	-	-
272	CVS, Na-channel block	FN	TN	TN	-	li ht	-	thyr ad; tes ad; adr bpha; adr bpha
77	CVS, PDE3 inhibitor	TN	TN	TN	adr	-	-	-
209	CVS, PDE3 inhibitor	FN	TN	TN	li ; kid	-	-	adr bpha
99	CVS, platelet aggregation inhibito	TN	NC	TN	-	li ht; thyr ht	-	-
282	CVS, platelet aggregation inhibito	FN	NC	FN	-	li ht	-	thyr ad; adr bpha; ut ac; li ad; ova ad; mam ad
63	CVS, remaining, 5-HT2 antagonist	TN	TN*	TN	spl ; li ; kid ; hrt ; pan ; br ; thy ; adr	-	-	-
141	CVS, remaining, B1 partial agonist	TN	TN*	TN	-	-	-	-
232	CVS, remaining, D1/alpha agonist	FN	TN*	TN	adr ; kid	-	-	pan ad
36	CVS, remaining, hemostatic	TN	TN*	TN	-	-	-	-
169	CVS, remaining, Hydrazinophtalzine	TP	TP*	TP	-	pit ht	thyr hp;	thyr ad; thyr ac

216	CVS, remaining, imidazole, PDE-inh	FN	TN*	TN	-	-	-	adr bpha
89	CVS, remaining, Nitr/K+ATP agonist	TN	TN*	TN	-	-	-	-
113	CVS, remaining, PDE5-inhibitor	TN	TN*	TN	-	li ht; thyr ht	-	-
225	CVS, remaining, Quinolone vasodila	FN	TN*	TN	li ; thyr ; adr ; spl ; pros ; tes	-	-	adr bpha
198	CVS, remaining, renin inhibitor	FN	TN*	TN	-	col ht	-	col ad; col ac
78	CVS, remaining, vasodilator	TN	TN*	TN	-	hrt ht	-	-
110	CVS, vasopressin-2 agonist	TN	TN	TN	-	-	-	-
131	CVS, vasopressin-2 agonist	TN	TN	TN	-	-	-	-
212	GI, 5HT4 agonist	FN	TN	TN	-	-	-	tes tu; pit ad
121	GI, 5HT4-agonist	TN	TN	TN	-	-	-	-
269	GI, 5HT4-agonist	FN	TN	TN	-	-	-	thyr ad; mam fad; pan ad; adr bpha; li ad; pit ad
48	GI, Histamine H2 antagonist	TN	TN	TN	br ; hrt ; kid ; tes ; li ; ova	-	-	-
94	GI, Histamine H2 antagonist	TN	TN	TN	li ; kid	-	-	-
210	GI, Histamine H2 antagonist	FN	TN	TN	li	-	-	tes ad
275	GI, Histamine H2 antagonist	FN	TN	TN	-	-	-	skin fibr
41	GI, Proton pump inhibitor	TN	TP	TP	-	-	-	-
178	GI, Proton pump inhibitor	TP	TP	TP	li ; li ; lu ; stom	li ht; stom ht; stom ht	stom hp	tes ad; tes ad
187	GI, Proton pump inhibitor	TP	TP	TP	li ; kid ; stom ; thyr ; hrt ; spl	li ht; stom ht; thyr ht	stom hp	adr bpha; tes ad; stom SCP; stom SCC; hsyst leu; pit ad
259	GI, Proton pump inhibitor	FN	TP	TP	-	stom ht	-	stom tu; stom SCC; li ad
119	GI, remaining, anti-osteoporose agent	TN	TN*	TN	-	-	-	-
32	GI, remaining, Fe-chelator	TN	TN*	TN	-	-	-	-

70	GI, remaining, Opioid, mu-agonist	TN	TN*	TN	-	-	-	-
30	GI, remaining, Phosphate binder	TN	TN*	TN	-	-	-	-
238	GI, remaining, Sugar alcohol	FN	TN*	TN	-	li ht	-	tes tu
80	GI, remaining, Synthetisch prostaglandin	TN	TN*	TN	adr ; li	-	-	-
215	HM, Dual 5 reductase inhibitor.	FN	TP	TP	-	-	-	tes ad
224	HM, Dual 5-reductase inhibitor	FN	TP	TP	-	-	-	thyr ad
221	HM, estrogen agonist	FN	TP	TP	-	-	-	pit ad
281	HM, estrogen agonist,	FN	TP	TP	-	-	-	li ad; mam ca
21	HM, GnRH agonist	TN	TP	TP	-	-	-	-
175	HM, GnRH agonist	TP	TP	TP	-	-	tes hp	pit ad
180	HM, GnRH agonist	TP	TP	TP	br	pit ht	pit hp	pit ad
254	HM, GnRH agonist	FN	TP	TP	-	-	-	adr bpha; adr mpha; islet ad; tes ad; pit ad; pit ca
286	HM, GnRH agonist	FN	TP	TP	-	-	-	pit ad; pit ca
42	HM, progestagen-estrogen contraceptive	TN	TP	TP	pit ; thyr	-	-	-
257	HM, progestagen-estrogen contraceptive.	FN	TP	TP	adr ; li	-	-	pit ad; mam ad; mam ac
214	HM, progesterone antagonist, birth cont	FN	TP	TP	li	-	-	li ad; ut ac; mam ac
166	HM, remaining, antiandrogen,	TP	TP	TP	tes ; adr	li ht; ova ht; adr ht; thyr ht	tes hp; ova hp	te ad; thyr ad; ut ac
241	HM, remaining, aromatase inhibitor	FN	TP	TP	-	li ht	-	ova gca; UGT pap
179	HM, selective estrogen modulator	TP	TP	TP	-	-	ova hp	kid ac; ova ad
201	HM, selective estrogen modulator	FN	TP	TP	-	-	-	kid ad; kid ac; ova ad
120	IS, Immunosuppressive	TN	TP	TP	-	-	-	-
140	IS, Immunosuppressive	TN	TP	TP	-	-	-	-

150	IS, Immunosuppressive	FP	TP	TP	-	-	ln hp	-
47	IS, Immunosuppressive, mTOR inhibitor	TN	TP	TP	-	thyr ht	-	-
152	IS, Immunosuppressive, mTOR inhibitor	FP	TP	TP	-	stom ht; thyr ht	stom hp	-
52	IS, Immunosuppressive, S1P antagonist	TN	TP	TP	-	-	-	-
8	IS, remaining	TN	NC	TN	-	-	-	-
242	IS, remaining, imidazothiazole derivative	FN	NC	FN	-	-	-	pit ad
76	MB, antidiabetic, alfa-glucosidase inhib	TN	TN	TN	-	-	-	-
194	MB, antidiabetic, alfa-glucosidase inhib	FN	TN	TN	-	-	-	tes ad; kid ad; kid ac;
68	MB, antidiabetic, DPP4 inhibitor	TN	TN	TN	-	thyr ht; li ht		-
111	MB, antidiabetic, DPP4 inhibitor	TN	TN	TN	-	-		-
114	MB, antidiabetic, DPP4 inhibitor	TN	TN	TN	-	-	-	-
139	MB, antidiabetic, DPP4 inhibitor	TN	TN	TN	-	-	-	
134	MB, antidiabetic, remaining, PPAR-gamma	TN	TP*	TP	hrt ; li	li ht	-	-
151	MB, Antidiabetic, remaining, SGLT-2 inhibitor	FP	TN*	TN	-	kid ht	kid hp	-
58	MB, Antidiabetic, remaining, SU derivative	TN	TN*	TN	-	-	-	-
46	MB, fibrate	TN	TP	TP	-	-	-	-
202	MB, fibrate	FN	TP	TP	-	-	-	tes tu; adr bpha; li ac
211	MB, fibrate	FN	TP	TP	li ; kid ; hrt ; adr ; tes	-	-	pan ad; stom tu; li ad; li ac
27	MB, HMG-CoA reductase inhibitor	TN	TP	TP	-	-	-	-
146	MB, HMG-CoA reductase inhibitor	FP	TP	TP	-	-	li hp	-

190	MB, HMG-CoA reductase inhibitor	TP	TP	TP	-	li ht	li hp; stom hp	ut polyp
173	MB, HMG-CoA-reductase inhibitor	TP	TP	TP	thyr	-	stom hp	stom SCP; thyr ac; thyr ad
267	MB, HMG-CoA-reductase inhibitor	FN	TP	TP	-	-	-	thyr ad; li ac
160	MB, remaining, 3 beta-hydroxysteroid de	FP	TN*	TN	-	adr ht	adr hp	-
130	MB, remaining, Aldose reductase inhibit	TN	TN*	TN	-	-	-	-
43	MB, remaining, hypertriglyceridemia	TN	TN*	TN	-	-	-	-
258	MB, remaining, Inhib.growth hormone	FN	TP*	TP	-	-	-	sk sar; ut ac
57	MB, remaining, lipid replacement	TN	TN*	TN	-	-	-	-
1	MB, remaining, nicotinic acid derived,	TN	TN*	TN	-	-	-	-
61	RS, Anticholinergic	TN	TN	TN	-	-	-	-
128	RS, Anticholinergic	TN	TN	TN	-	-	-	-
191	RS, Beta2 agonist	TP	TP	TP	-	-	nose hp	ova leio; pit ad
228	RS, Beta2 agonist	FN	TP	TP	-	pan ht	-	thyr ad; thyr ac; ova leio; mam ac
280	RS, Beta2 agonist	FN	TP	TP	-	-	-	ova leio
288	RS, Beta2 agonist	FN	TP	TP	lu ; hrt	hrt ht	-	ova leio; pit ad; pit ac
199	RS, Beta2-agonist	FN	TP	TP	li	-	-	thyr ad
168	RS, Corticosteroid	TP	TP	TP	-	-	mam hp	mam fad; li ac; br astr; li ad
170	RS, Corticosteroid	TP	TP	TP	many; tes ; br ; hrt ; kid ; pit ; li	li ht	pan hp; ln hp	pan ad; pan ac; bo most; li ad; li ac; li ac; mam ad; mam ac
227	RS, Corticosteroid	FN	TP	TP	-	-	-	islet tu; adr bpha; skin sar
11	RS, Histamine H1 antagonist	TN	TN	TN	-	-	-	-
12	RS, Histamine H1 antagonist	TN	TN	TN	li ; lu ; hrt ; kid ; tes	li ht	-	-
154	RS, Histamine H1 antagonist	FP	TN	TN	-	-	mam hp	-
155	RS, Histamine H1 antagonist	FP	TN	TN	li	li ht	pan hp	-

195	RS, Histamine H1 antagonist	FN	TN	TN	li ; kid	-	-	adr bpha
207	RS, Histamine H1 antagonist	FN	TN	TN	-	li ht	-	thyr ad; pit ac; li ac
67	RS, HistamineH1 antagonist	TN	TN	TN	-	li ht	-	-
184	RS, remaining, antifibrotic	TP	NC	TN	-	adr ht	adr hp	li ad; ut ac
82	RS, remaining, Leukotriene receptor a	TN	TN	TN	-	-	-	-
116	RS, remaining, Mest cell stabilisor	TN	TN	TN	-	-	-	-
264	RS, remaining, Methylxanthine-derivate	FN	TN	TN	li	-	-	tes tu; mam fad
31	UB, Anticholinergic	TN	TN	TN	-	-	-	-
51	UB, Anticholinergic	TN	TN	TN	-	-	0	-
268	UB, Anticholinergic	FN	TN	TN	-	li ht	-	ut polyp; kid pap
283	UB, Anticholinergic	FN	TN	TN	-	-	-	kid sar
287	UB, Anticholinergic	FN	TN	TN	-	-	-	skin sar
125	UB, Anticholinergic and calcium antagoni	TN	TN	TN	thyr ; adr ; ova ; li	-	-	-
153	UB, remaining xanthine oxidase inhibito	FP	TN*	TN	-	-	thyr hp	-
79	UB, remaining,oral Beta 3 agonist	TN	TN*	TN	-	li ht	-	-
62	ZZ, remaining, CFTR potentiator	TN	TN*	TN	-	-	-	-
39	ZZ, Remaining, Prostaglandin E2	TN	TN*	TN	-	-	-	-
109	ZZ, remaining, protein kinase C-beta inhibitor	TN	TN*	TN	-	-	-	-
196	ZZ, Remaining, retinoid, topical, keratinocyte	FN	TN*	TN	pit ; adr	-	-	adr bpha; thyr ad
